# Parvoviruses Cause Nuclear Envelope Breakdown by Activating Key Enzymes of Mitosis

**DOI:** 10.1371/journal.ppat.1003671

**Published:** 2013-10-31

**Authors:** Manvi Porwal, Sarah Cohen, Kenza Snoussi, Ruth Popa-Wagner, Fenja Anderson, Nathalie Dugot-Senant, Harald Wodrich, Christiane Dinsart, Jürgen A. Kleinschmidt, Nelly Panté, Michael Kann

**Affiliations:** 1 Institute of Medical Virology, University of Giessen, Giessen, Germany; 2 Univ. de Bordeaux, Microbiologie fondamentale et Pathogénicité, UMR 5234, Bordeaux, France; 3 CNRS, Microbiologie fondamentale et Pathogénicité, UMR 5234, Bordeaux, France; 4 Department of Zoology, University of British Columbia, Vancouver, British Columbia, Canada; 5 German Cancer Research Center, Heidelberg, Germany; 6 Institute of Virology, Hannover Medical School, Hannover, Germany; 7 Inserm U889, Univ. de Bordeaux 2, Bordeaux, France; 8 Inserm U701, German Cancer Research Center, Heidelberg, Germany; 9 CHU de Bordeaux, Bordeaux, France; King's College London School of Medicine, United Kingdom

## Abstract

Disassembly of the nuclear lamina is essential in mitosis and apoptosis requiring multiple coordinated enzymatic activities in nucleus and cytoplasm. Activation and coordination of the different activities is poorly understood and moreover complicated as some factors translocate between cytoplasm and nucleus in preparatory phases. Here we used the ability of parvoviruses to induce nuclear membrane breakdown to understand the triggers of key mitotic enzymes. Nuclear envelope disintegration was shown upon infection, microinjection but also upon their application to permeabilized cells. The latter technique also showed that nuclear envelope disintegration was independent upon soluble cytoplasmic factors. Using time-lapse microscopy, we observed that nuclear disassembly exhibited mitosis-like kinetics and occurred suddenly, implying a catastrophic event irrespective of cell- or type of parvovirus used. Analyzing the order of the processes allowed us to propose a model starting with direct binding of parvoviruses to distinct proteins of the nuclear pore causing structural rearrangement of the parvoviruses. The resulting exposure of domains comprising amphipathic helices was required for nuclear envelope disintegration, which comprised disruption of inner and outer nuclear membrane as shown by electron microscopy. Consistent with Ca^++^ efflux from the lumen between inner and outer nuclear membrane we found that Ca^++^ was essential for nuclear disassembly by activating PKC. PKC activation then triggered activation of cdk-2, which became further activated by caspase-3. Collectively our study shows a unique interaction of a virus with the nuclear envelope, provides evidence that a nuclear pool of executing enzymes is sufficient for nuclear disassembly in quiescent cells, and demonstrates that nuclear disassembly can be uncoupled from initial phases of mitosis.

## Author Summary

Parvoviruses are small non-enveloped DNA viruses successfully used in gene therapy. Their nuclear replication requires transit of the nuclear envelope. Analyzing the interaction between parvoviruses and the nucleus, we showed that despite their small size, they did not traverse the nuclear pore, but attached directly to proteins of the nuclear pore complex. We observed that this binding induced structural changes of the parvoviruses and that the structural rearrangement was essential for triggering a signal cascade resulting in disintegration of the nuclear envelope. Physiologically such nuclear envelope breakdown occurs late during prophase of mitosis. Our finding that the parvovirus-mediated nuclear envelope breakdown also occurred in the absence of soluble cytosolic factors allowed us to decipher the intra nuclear pathways involved in nuclear envelope destabilization. Consistently with the physiological disintegration we found that key enzymes of mitosis were essential and we further identified Ca^++^ as the initial trigger. Thus our data not only show a unique pathway of how a DNA virus interacts with the nucleus but also describes a virus-based system allowing the first time to analyze selectively the intranuclear pathways leading to nuclear envelope disintegration.

## Introduction

The nuclear envelope separates cytoplasm and nucleus requiring shuttling of cargos between the compartments. In non-dividing cells macromolecule exchange occurs via the nuclear pore complexes (NPC), which are composed of ∼30 different proteins (nucleoporins, Nups). NPCs allow the passage of macromolecules only in complex with soluble transport receptors as e.g. the nuclear import receptors of the importin (karyopherin) ß superfamily [1]. During transport the receptors interact with those nucleoporins comprising FxFG repeats, which are localized on unstructured domains [2]. At the end of nuclear import this complex becomes dissociated by the small GTPase Ran in its GTP-bound form. While the cargo diffuses deeper into the karyoplasm, the receptor-RanGTP complex is exported into the cytoplasm [3]. The nuclear envelope is composed of the double lipid bilayer of outer nuclear membrane (ONM) and inner nuclear membrane (INM) and a matrix of proteins separating INM and the chromatin. The matrix is composed of both peripheral and integral membrane proteins, including lamins and lamin-associated proteins. The nuclear lamina is required for proper cell cycle regulation, chromatin organization, DNA replication, cell differentiation, and apoptosis [4]. In contrast to closed mitosis in yeast open mitosis as it is the case in other eukaryotes but also apoptosis requires that the nuclear envelope (NE) disassembles (nuclear envelope breakdown, NEBD), involving depolymerization of the lamin network. In mitosis, NEBD starts at a single hole in the nuclear envelope, which expands within minutes over the nuclear surface [5]. As the space between ONM and INM in continuation with ER lumen is the space where free Ca^++^ is stored increased perinuclear Ca^++^ is observed directly before the NE disintegrates [6]. In contrast NEBD in apoptosis is characterized by dynamic nuclear membrane blebbing and fragmentation [7].

Several enzymatic activities participate in NEBD. In mitosis, lamin depolymerization is executed by hyper phosphorylation of lamin A/C, B1, B2 comprising different protein kinase C isoforms and cyclin-dependent kinases (cdks); their balanced activities control G1/S transition [8]. The role of caspase-3 in mitosis is controversial [9]–[11]. NEBD in apoptosis requires PKCδ and cdks but nuclear dismantling depends on caspase-3 [12].

NEBD is tightly controlled by the cdks and PKC isoenzymes activities. Their balanced activities controls G1/S and G2/M transitions and links signal transduction pathways to the cell cycle machinery [13]. Several reasons complicate research on NEBD: the regulations and interactions are complex and the executing enzymes - as it was described for PKC α/δ and caspase-3 - become imported into the nucleus during the initial phases of apoptosis or mitosis [14]–[16] where they fulfil other functions as for instance lamin phosphorylation and degradation than in the cytoplasm.

Parvoviruses (PV) are well conserved viruses, comprising dependo-viruses as the adeno-associated virus (AAV), and autonomous PV as the canine parvovirus and H1. PVs are used in gene therapy trials and AAV-based vectors were recently licensed for gene therapy of lipoprotein lipase deficiency. Parvoviruses are composed of two (three in AAV) co-terminal structural proteins, VP1 and VP2, which form a capsid of 26 nm in diameter. The larger protein (VP1) has an additional/unique N terminal sequence (VP1u) comprising a potential nuclear localization signal (NLS) and a phospholipase A_2_ (PLA_2_) activity, which is essential for infection [17]. VP1u is hidden within the virion but is predicted to become exposed during infection [18], [19] while the capsid remains intact. PVs enter the cell by endocytosis and are subsequently transported in endosomes towards the nuclear periphery [20]. Acidification is required for infection and only a small proportion of PV escape the late endosomes [21], predicted to be mediated by the PLA_2_ domain on VP1u. PVs contain a single stranded DNA genome, which is replicated by cellular DNA polymerases inside the cell nucleus. DNA release remains poorly understood but occurs without capsid disassembly [22] and at least AAV2 enters the nucleus fully assembled according to the majority of studies (e.g. [23], [24]).

The interaction between PV and nuclear envelope are not fully understood. After microinjection of canine PV into the cytosol, capsids appear in the nucleus after hours [25]. It remains open if these capsids were derived from nuclear import of the microinjected capsids or from progeny capsid formation. Microinjection of Minute Virus of Mice (MVM) into *Xenopus laevis* oocytes cause distinct breaks of the nuclear envelope [26], [27], which could be large enough to allow entry of the PV capsids into the nucleus [28].

We investigated the interaction between PV and the nucleus in more detail finding that PV attached directly – without the need of nuclear import receptors - to the NPC, which activated an intranuclear cascade leading to degradation of the nuclear envelope.

## Results and Discussion

### Infection of HeLa cells with high MOI of parvovirus H1 causes local disintegration of the nuclear envelope

**Figure 1:**
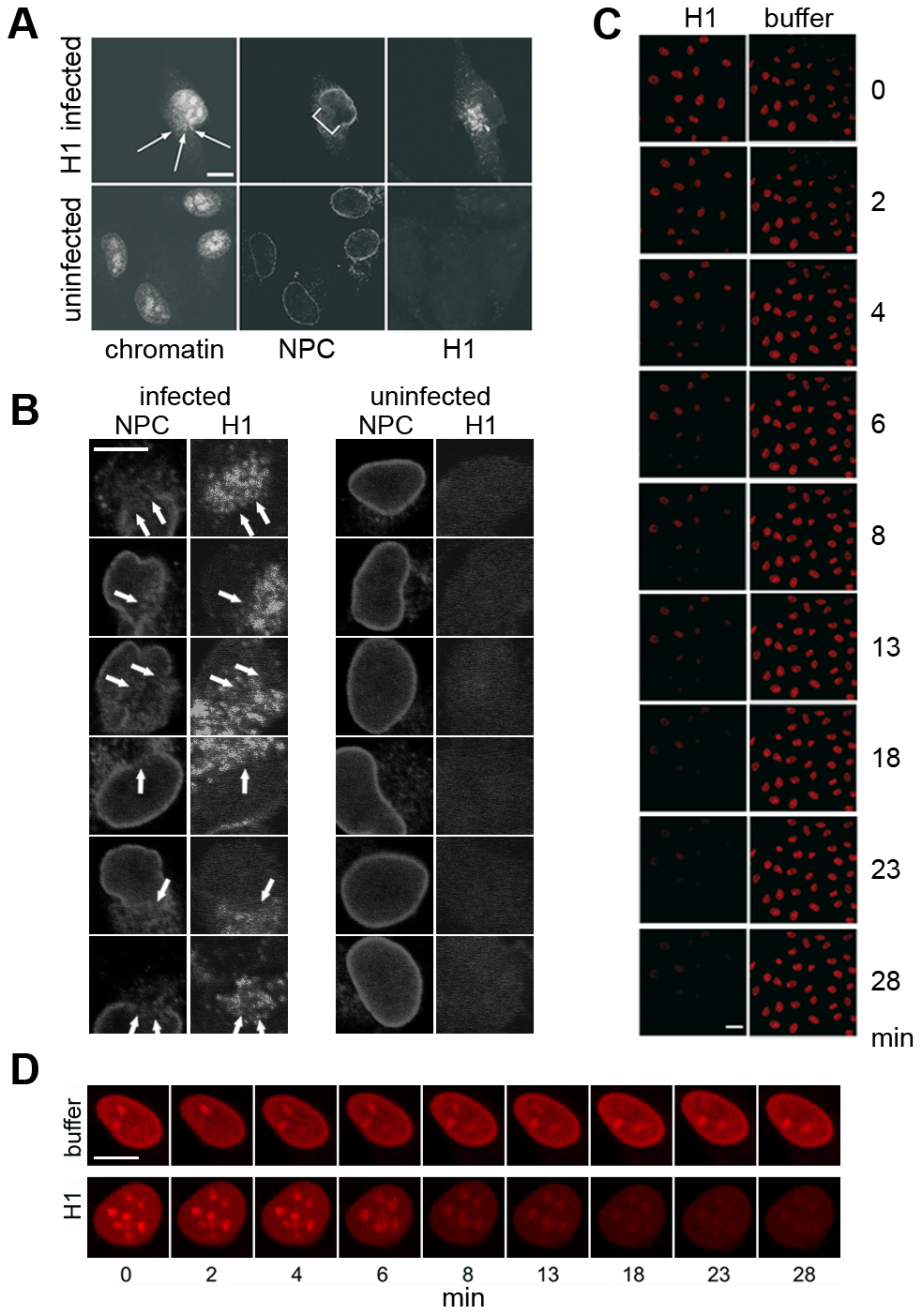
Parvovirus H1 cause NEBD with chromatin escape. **A, B:** Infection of HeLa cells with 1000 H1 per cell. Confocal laser scan microscopy after indirect immune fluorescence. Bar = 10 µm. Chromatin was stained by PI, NPCs by mAb414 and H1 by anti VP2 antibodies. **A.** Example of a cell in which chromatin passes through the membrane break into the cytoplasm. The NE break is indicated by a bracket, the extruding chromatin is indicated by arrows. **B.** Magnification of nuclear envelope breaks observed in different cells. Left columns show H1 infected HeLa cells, the right columns uninfected cells. The NPC stain by mAb414 and the H1 stain by anti VP2 antibodies are indicated on top. The arrows indicate the nuclear envelope breaks observed in 102 out of infected cells 913 cells (11%). Uninfected cells showed these discontinuities in only 2 out of 294 cells. Together the results indicate that H1 infection causes local NEBD causing holes partially large enough to allow passage of host chromatin into the cytoplasm. Bar = 10 µm. **C, D.** Time lapse microscopy of permeabilized HeLa cells incubated in buffer in the absence or in the presence of 300 H1 per permeabilized cell. No soluble cytosolic factors were present in the reaction. Images were taken by confocal microscopy at nine time points in order to exclude bleaching of the chromatin fluorescence by PI. The time points are indicated right (C) or below (D) of the panels. **C.** Overview of chromatin disappearance in nuclei in one microscopical field. Left column: H1, right column: buffer only. Bar = 20 µm. **D.** Magnification of chromatin disappearance in one HeLa cell. Bar 0 10 µm. Images C and D show that the PI-stained chromatin was lost in permeabilized HeLa cells, corresponding to the NE breaks observed upon infection despite of the absence of cytosol. No perinuclear chromatin patches as in apoptosis appeared.

Upon infection of 1000 genome-containing PV H1 per cell we observed local NEBD at the site where PV accumulated. NEBD was indicated by the loss of NPC stain and – in some cases - chromatin escape into the cytoplasm (Fig. 1A). Nuclear envelope disintegrations were observed in 11% of the infected cells (102 out of 913) at a time before progeny viruses are made. In non-infected cells <1% of cells (2/294) showed such damage indicating that the phenomenon was parvovirus-dependent. This assumption was supported by the observation that disintegration only occurred at those sites of the nucleus were parvoviruses accumulated (Fig. 1B). We did neither observe chromatin condensation as it occurs in prophase of mitosis nor that we monitored the formation of chromatin patches closed to the NE as in apoptosis. Chromatin fragmentation, yet another characteristic of apoptosis was also not observed. Similar local disruptions were however observed recently upon egress of cytomegalovirus capsids [29] and also – as a temporary phenomenon - for MVM [27].

### Nuclear envelope disintegration in digitonin-permeabilized cells

To investigate virus-induced nuclear disassembly in more detail we analysed the PV-mediated NEBD by digitonin-permeabilized HeLa cells using confocal laser scanning microscopy. Digitonin permeabilizes cholesterol-containing membranes leaving the nuclear and ER membrane intact [30]. Permeabilization was stopped by washing the cells at 4°C prior to destruction of the part of the plasma membrane, which connects the permeabilized cell with the cover slip. At 4°C microtubules depolymerise and the washes remove soluble cytoplasmic proteins including nuclear transport receptors. Accordingly we did not observe active nuclear import after addition of a karyophilic cargo (Supporting Information, Fig. S1A) and α tubulin was reduced to 2% compared to unpermeabilized cells (Supporting Information, Fig. S1B). We thus concluded that permeabilized, washed cells are devoid of significant amounts of soluble cytosolic proteins including nuclear import receptors.

We added H1 to the permeabilized cells in the absence of cytosolic factors but in which cellular chromatin was stained by propidium iodide (PI), allowing to record nuclear integrity by time-lapse microscopy. Figure 1C shows that chromatin fluorescence disappeared upon addition of H1. As in infected cells no significant chromatin condensation was found, which is exemplified in figure 1D. Only little loss of fluorescence (4% in average) was observed in nuclei to which buffer was added, probably due to bleaching of the stain upon illumination with the laser beam. Loss of fluorescence was dye-independent as the same results were obtained upon chromatin stain using DAPI (see below), implying that the loss of stain was based on chromatin escape.

**Figure 2:**
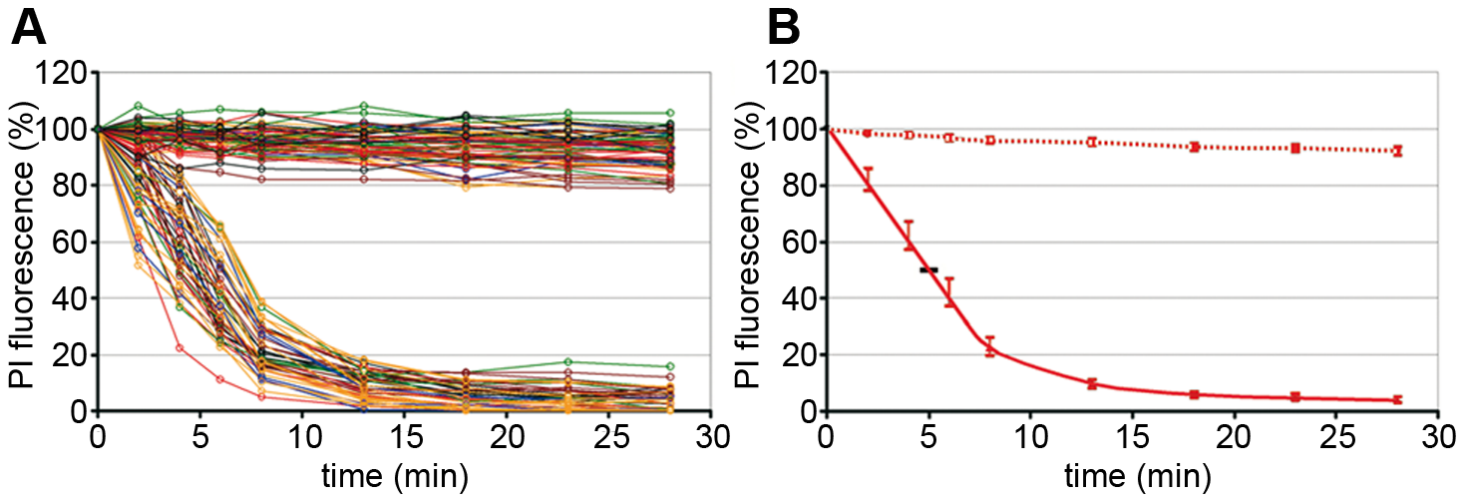
Intra and inter assay variability of chromatin loss in permeabilized HeLa cells incubated with or without H1. Merge of six experiments. The chromatin by PI was quantified in 42 nuclei exposed to H1 and in 67 nuclei exposed to buffer only. **A.** Each circle represents the relative PI fluorescence at the indicated time. The fluorescence at t = 0 of each cell was set as 100%. The lines connect the fluorescence of one nucleus at different times. Each colour represents one experiment. Y-axis: relative fluorescence in %, x-axis: time in min. **B.** Same data as in A. but showing the mean fluorescence and the 95% confidence intervals (CI) (bars). The horizontal bar shows the 95% CI for the 50% loss of PI fluorescence (4.6 to 5.4 min). The data show that parvovirus-mediated NEBD exhibits small variations between different assays and between individual nuclei.

Quantification of the chromatin escape in several assays was highly reproducible but varied between individual nuclei (Fig. 2A). The distribution of the fluorescence loss showed a Gaussian normal distribution (not shown), which allows presentation of the results as mean values with 95% confidence intervals (95% CI) shown in figure 2B. In fact non-overlapping CIs depict statistically significant differences. Figure 2B further shows that 50% of fluorescent chromatin was lost in mean within 4.6 to 5.4 min after PV addition, which is in the same range as the fenestration during mitosis [31] and much faster than nuclear degradation in apoptosis, which takes hours [32].

### Characterization of PV and NEBD

The kinetic of chromatin escape was dependent upon the number of H1 (Supporting Information, Fig. S2A) but independent upon the viral purification protocol (Supporting Information, Fig. S2B). A mock purification from non-infected cells did not show any chromatin escape (Fig. S2B) suggesting that chromatin escape was viral dose-dependent but not caused by cellular components co-sedimenting with H1. To further exclude that cellular proteins interacting with the parvoviruses were co-purified causing nuclear disintegration we analyzed the purified parvoviruses by silver stain after SDS PAGE. Figure S2C (Supporting Information) shows that all bands detectable by the silver stain also reacted with a polyclonal anti parvovirus H1 antibody, indicating that the preparation after iodixanol gradient centrifugation was free of contaminating cellular proteins. The parvovirus H1 preparation, which was purified via a CsCl gradient, was used for crystallization and the purity analysis was published elsewhere [33].

We observed that incubation of the permeabilized cells with H1 at room temperature instead of 37°C decelerated nuclear disintegration by 5fold (Supporting Information, Fig. S3A), which is in accordance with reduced enzymatic activities at lower temperatures. This observation makes it unlikely that parvovirus H1 has caused nuclear envelope damage by direct physical interaction. In accordance with this conclusion we observed an entire inhibition of H1-mediated nuclear disintegration when the permeabilized cells were energy-depleted (Supporting Information, Fig. S3B).

**Figure 3:**
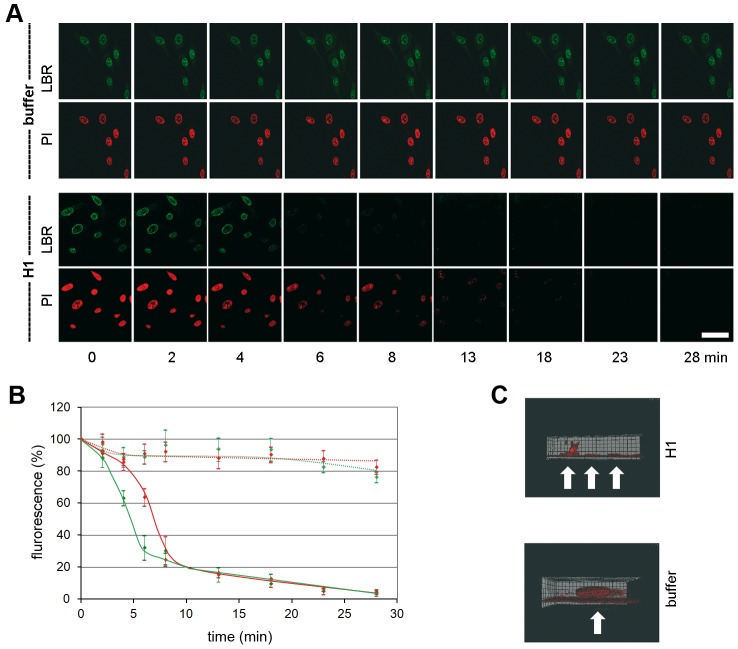
Loss of chromatin and lamin B receptor (LBR) upon H1 exposure to permeabilized NRK cells. Per permeabilized cell 300 H1 were added. **A.** Green: EYFP-LBR, red PI-chromatin. Bar = 20 µm. **B.** Quantification of A. The bars depict 95% CIs. Green dotted line: LBR in buffer only cells (n = 9); red dotted line: PI in buffer only cells. Green line: LBR with H1 (n = 9); red line: PI with H1. The figures A and B show that LBR dissociates first from the nuclear envelope before chromatin becomes released. **C.** 3D reconstruction of PI stained nuclei 15 min after addition of H1 (top) or buffer (bottom). The cover slip is at the bottom of the images, the cell surface on top. Cells are indicated by white arrows. The panels show that at in some cells after 15 min some chromatin stayed attached to the nuclear membrane adjacent to the part of plasma membrane, which is attached to the cover slip.

The loss of chromatin requires not only pore formation of the nuclear membranes but also the disassembly of the nuclear lamina as it occurs upon NEBD. We analyzed the disintegration of the lamina in normal rat kidney cells (NRK cells), expressing the enhanced yellow fluorescent protein fused to the lamin B receptor (LBR). LBR localizes at the inner nuclear membrane and anchors the lamina and the heterochromatin to the membrane. Addition of H1 to permeabilized NRK cells – again in the absence of cytosolic factors – led to rapid loss of LBR, which was significantly faster than the loss of PI fluorescence (50% LBR: 5.0 min, PI: 6.6 min, Fig. 3A, B). Blebbing of the nuclear envelope, as it occurs in apoptosis, was not observed.

Confocal laser scanning microscopy limits the observation to the equatorial section of the nuclei. Thus we next analyzed chromatin distribution after H1 exposure to permeabilized HeLa cells by 3D reconstruction. Due to bleaching, which occurs during multiple scans needed for the reconstruction we restricted our analysis to one time point 15 min after addition of H1. At this time little fluorescence rested in the equatorial sections. Figure 3C confirmed the absence of chromatin in most cells but also showed one cell in which some chromatin stayed attached to that area of nuclear membrane, which was directly in front of the plasma membrane attaching to the cover slip. As HeLa cells are extremely dedifferentiated a polarization-dependent effect was unlikely. Instead we hypothesize that the PV had restricted access to this area of the NE connecting the nucleus with the cover slip-bound region of plasma membrane. This idea is supported by the observation that digitonin permeabilization starts on the plasma membrane accessible to the exterior before progressing to the plasma membrane underneath the nucleus indicating an area of restricted access even for smaller molecules. Further this model is consistent with our observation that H1 caused local NEBD in infection during which a locally increased concentration of PV was observed (Fig. 1B).

### Nuclear envelope disintegration is conserved amongst different parvoviruses

**Figure 4:**
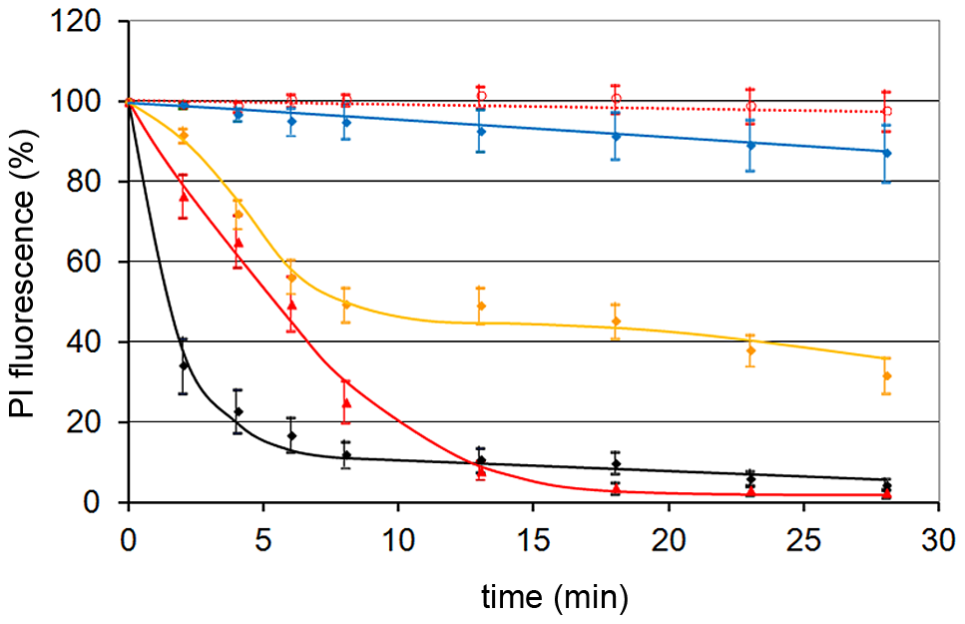
NEBD capacity of different PV. Per permeabilized HeLa cell 300 of each PV were added. The figure shows the quantification of the PI stain as in figure 2B, and the 95% CIs at each time point. Red dotted line: buffer only (n = 14). Blue line: AAV2 (n = 13), orange line: canine parvovirus (n = 35), red line: H1 (n = 23), black line: AAV2 after acidification to pH 5.2 and subsequent neutralization (n = 10). Acidification to pH 5.2 of H1 did not change the NEBD activity (not shown). The figure shows that NEBD is independent upon the type of PV with a similar kinetic but to a different extent. Acidification was needed for AAV but not for H1 and the canine parvovirus.

Having shown that NEBD is cell type-independent we next asked if this phenomenon is also conserved between different PV. We analyzed the canine parvovirus and AAV2 showing that all of them disintegrated the nuclear envelope (Fig. 4A) leading to chromatin release from the nuclei of permeabilized HeLa cells. The kinetic of NEBD were similar although AAV2 was somewhat more efficient than H1 while the canine parvovirus showed a delayed disintegration. As for H1 no soluble cytosolic factors were needed but AAV2-mediated NEBD was limited to capsids, which were exposed to pH 5.2 (and subsequently neutralized). In fact pH 5.2 is the characteristic pH of late endosomes from which PVs escape [20] but the acidification of H1 (not shown) had no effect indicating discrete differences between both viruses.

### Parvoviruses need interaction with the nuclear pore complex to cause NEBD

**Figure 5:**
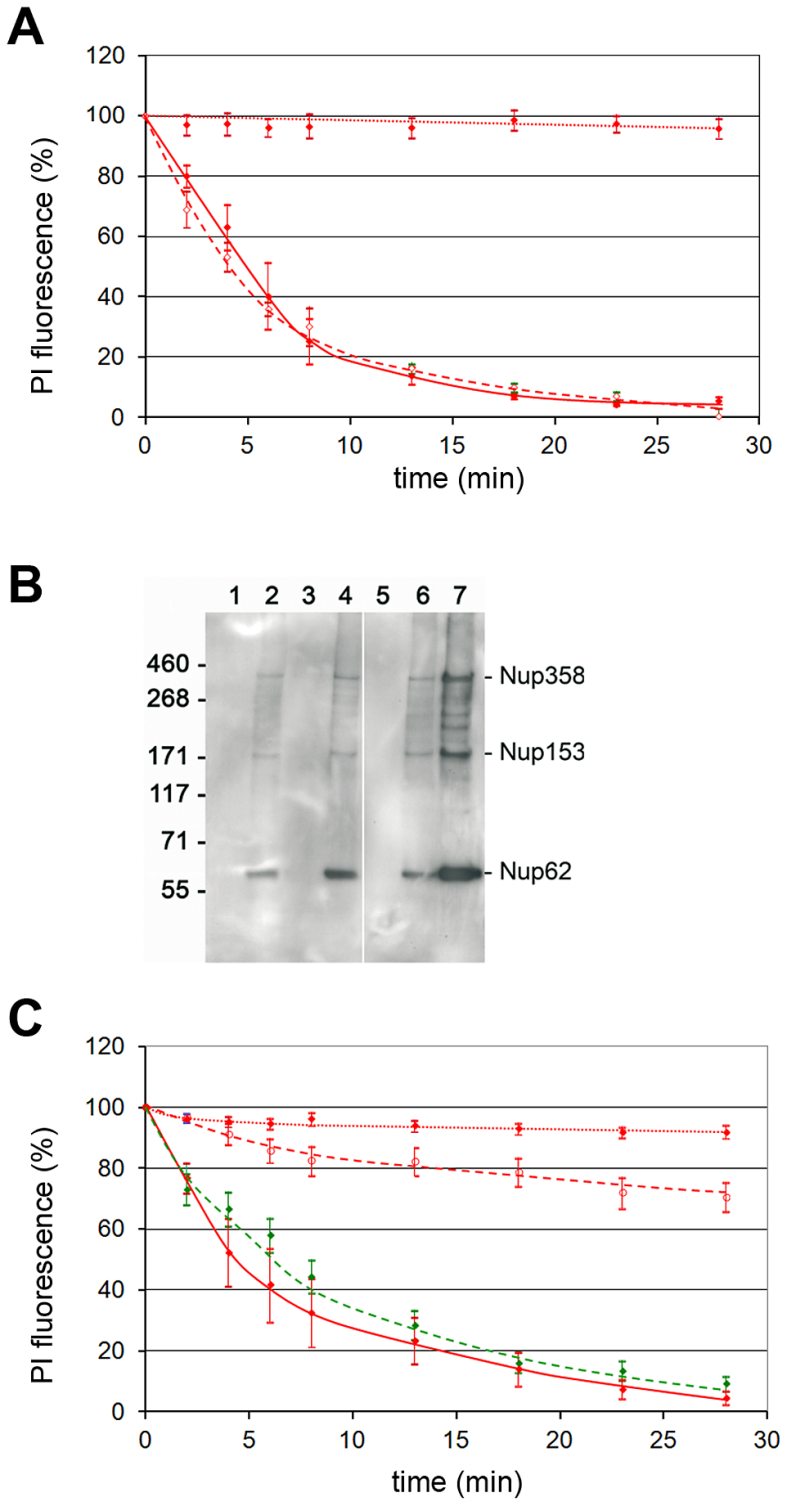
PV interact directly with nucleoporins required for NEBD. **A.** WGA does not inhibit NEBD upon addition of 300 H1 per permeabilized cell. The figure shows the quantification of the PI stain as in figure 2B, and the 95% CIs at each time point. Red dotted line: buffer only (n = 8), red line: H1, green dashed line: H1 in the presence of 1 mg/ml WGA (n = 4). **B.** Parvoviruses bind directly to nucleoporins. Co-precipitated Nups were detected by Western blot using the mAb414, which interacts with different FG repeat-containing Nups including Nup358, 214,153 and 62. The MW is given on the left, the Nups are indicated on the right. 1: AAV2 (acidified and neutralized) without Nups, 2: same AAV2+Nups, 3: H1, 4: H1+Nups, 5: beads+Nups, 6: 12 µg, 7: 36 µg Nups directly loaded on the gel. Nup153 migrates at ∼170 kDa, as it was described elsewhere [70]. **C.** Blocking the NPCs by hepatitis B virus capsids inhibits H1-mediated NEBD. Conditions and read-out as in A. Red dotted line: buffer only (n = 14), red dashed line: H1 after pre-incubation of the NPCs with an excess (1200 ng) of *in vitro* phosphorylated capsids of the hepatitis B virus in the presence of transport receptors (n = 18), green dashed line: same treatment with a mutant of the hepatitis B virus capsid, which lacks the C terminus that is need for NPC interaction (n = 9), red line: H1 (n = 7).

To identify the trigger of NEBD we analyzed the contact site for PV at the nuclear envelope. Addition of wheat germ agglutinin, which blocks the attachment of nuclear import receptors to NPCs and which prevents active nuclear import at this concentration [34], did not prevent NEBD (Fig. 5A). This finding is in agreement with observations in *Xenopus laevis* oocytes in which wheat germ agglutinin has no effect on MVM-mediated pore formation [27]. Cohen et al. concluded that NPCs are not involved in nuclear envelope degradation we considered a direct interaction of the PV capsids with the proteins of the nuclear pore. In fact this hypothesis is consistent with our observation that soluble cytosolic proteins were not required for PV-mediated NEBD. We thus performed coprecipitations of H1 and AAV2 (after acidification) using a purified preparation of Nups (Supporting Information, Fig. S4). The preparation was devoid of importin α and importin β was reduced by 50fold compared to intact cells, which is consistent with the observation that purified nuclei - the first step of Nup preparation - are incompetent for active nuclear import. The changed abundance of the Nups in the preparation compared to intact cells further indicated that the NPCs were dissociated into Nups upon preparation.

Despite of the absence of importin α both AAV2 after acidification and H1 precipitated Nups 358, 153 and 62 (Fig. 5B). Nup62 gave the strongest band likely due to its higher abundance in NPCs than Nup358 and Nup153 [35]. In fact the strength of the signals corresponded well to those present in the preparation suggesting that there is no preferential interaction of the PV with one of the Nups. However as the antibody used for detection is limited to some FXFG repeat-containing Nups [36] we cannot exclude that other Nups were precipitated. Nonetheless the observation that the Nups were dissociated upon their purification argues against a co-precipitation of the Nups based on PV interaction to one Nup only, which than forms a complex with the other Nups. Furthermore the only known factor interacting with all PV-precipitated Nups would be the complex of importin α andβ, which could bind to the NLS on VP1u. The absence of importin α makes this scenario unlikely and supports direct interaction of the Nups with H1 and AAV2.

For getting direct evidence that PV – NPC interactions are required for NEBD we next preloaded the NPCs of permeabilized cells with an excess of hepatitis B virus capsids, which attach to Nup153 without becoming released into the nucleus [37]. The capsids were bound in the presence of a cytosolic lysate, which contains the required nuclear transport factors importin α and β, needed to attach the HBV capsid to the NPC. At the NPC, the transport receptors are dissociated leading to direct attachment to Nup153. Consequently, the capsids localize at the cytoplasmic and nuclear phase of the NPC. After removal of the lysate by washing we added H1 observing that capsid-saturation blocked NEBD nearly entirely (Fig. 5C). Inhibition was specific as a capsid mutant, which fails to bind the transport receptors, thus not interacting with the nuclear pore, did not interfere with NEBD (Fig. 5C).

To further exclude NPC-independent interaction with membranes we incubated PV at the same concentration of PV used in permeabilized cells with intact cells together with PI for 15 min, showing that the membrane impermeable PI remained excluded from the cells (Supporting Information Fig. S5). In summary we conclude that PVs need direct attachment to NPCs for membrane degradation.

Considering that we added 300 H1 per nucleus, which is not sufficient to saturate the approx. 5000 NPCs per HeLa cells [37] is was surprising that PV cause discontinuities large enough to allow the escape of chromatin. We thus hypothesized that the holes spread as in mitosis [5].

### VP1u becomes exposed upon Nup interaction and is needed for NEBD

**Figure 6:**
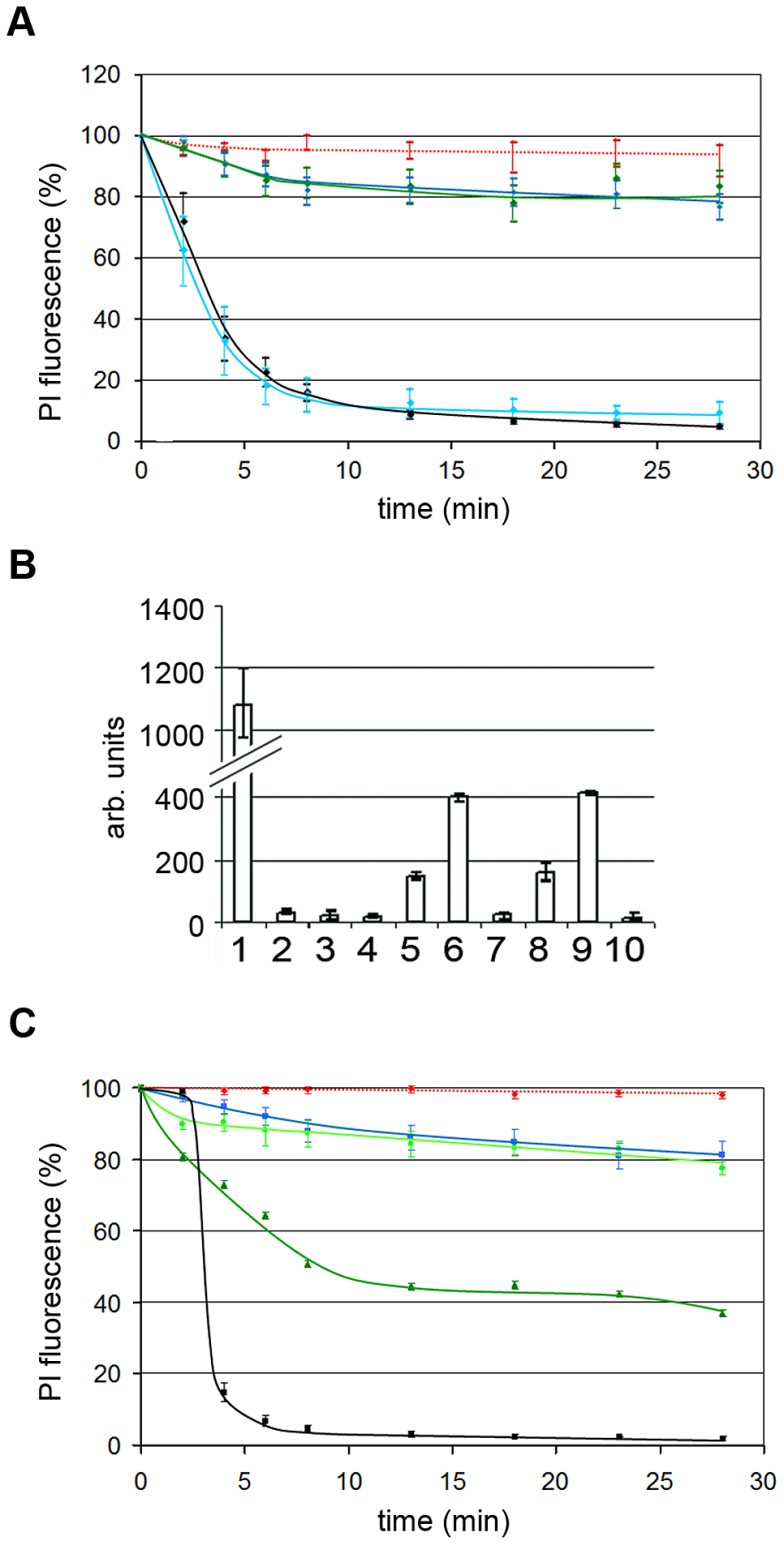
A. Impact of VP1u on NEBD. Quantifications of the PI fluorescences with the mean values and the 95% confidence intervals (bars) after addition of 300 genome-containing wt AAV2 particles and 300 mutant AAV2 per permeabilized HeLa cell. Red dotted line: buffer only (n = 15), blue line: mt AAV2 ΔVP1 (n = 14), blue line: wt AAV2 (not acidified) (n = 20), black line: wt AAV2 (acidified) (n = 11), green line: mt AAV2 ΔVP2 (acidified; n = 18). The panel shows that VP1 is indispensable for NEBD for AAV2. **B.** PV PLA_2_ activity increases upon interaction with nucleoporins. 1. Positive control (PLA_2_ from bee venom), 2. H1, 3. AAV2, 4. AAV2 after acidification, 5. H1+nucleoporins, 6. H1+nucleoporins+Ca^++^, 7. AAV2+nucleoporins, 8. AAV2 after acidification+nucleoporins, 9. AAV2 after acidification+nucleoporins+Ca^++^, 10. Nucleoporins. Y-axis: arb. units. The observations indicate that interaction with nucleoporins cause PLA_2_ activation, which indicates the exposure of VP1u on the surface of the particles. **C.** PLA_2_ is not essential for NEBD. Quantification of PI fluorescence as in A. Red dotted line: buffer; black line: AAV2 wt (n = 10), pH-treated; light green line: AAV2 HD/AN mt (n = 10); green line: AAV2 HD/AN mt acidified and neutralized (n = 10). Wt AAV2: 300 per permeabilized cell; mt viruses 120 per permeabilized cell. Collectively the three panels show that PV need VP1 for NEBD and that VP1u becomes exposed upon Nup interaction. The PLA_2_ activity on VP1u is however not required.

Asking which PV domain causes NEBD, we added two mutants of AAV2 to permeabilized cells: AAV2-ΔVP1 is devoid of VP1 including VP1u and AAV2-ΔVP2, which is devoid of VP2 but which comprises VP1u [38]. NEBD was restricted to the VP1u-comprising mutant (Fig. 6A).

The need of VP1u, which normally is hidden in the viral context for NEBD, suggests that the attachment to the nucleoporins causes structural changes including exposure of VP1u. We determined VP1u-exposure using the PLA_2_ domain, which becomes only active upon externalization for instance after heat treatment and acidification (PV B19; [39]). Incubating purified Nups with H1 increased the PLA_2_ activity by ∼2.5 fold (Fig. 6B) indicating exposure of VP1u upon nucleoporins association. AAV2 needed acidification prior to Nup addition in order to exhibit PLA_2_ activity. This finding corresponds to the acidification-dependent NEBD capacity of AAV2 and supports the need of the structural change for the phenomenon.

The PLA_2_ domain present on VP1u could not only be involved in endosomal escape as it was described recently [18] but also in NEBD. We thus added an AAV2 mutant, in which the catalytic centre of the PLA_2_ domain was inactivated [38], to permeabilized cells. As shown in figure 6C the mutant caused NEBD as wild type AAV2 only after exposure to acidic pH excluding that PLA_2_ activity is needed in NEBD. The effect was not as pronounced as for wt AAV2 as the mutant could not be obtained in the same concentration (only 40%). Additional investigations are currently performed for identifying the responsible domain on VP1u.

### Electron microscopy of pore formation in the nuclear envelope by H1

**Figure 7:**
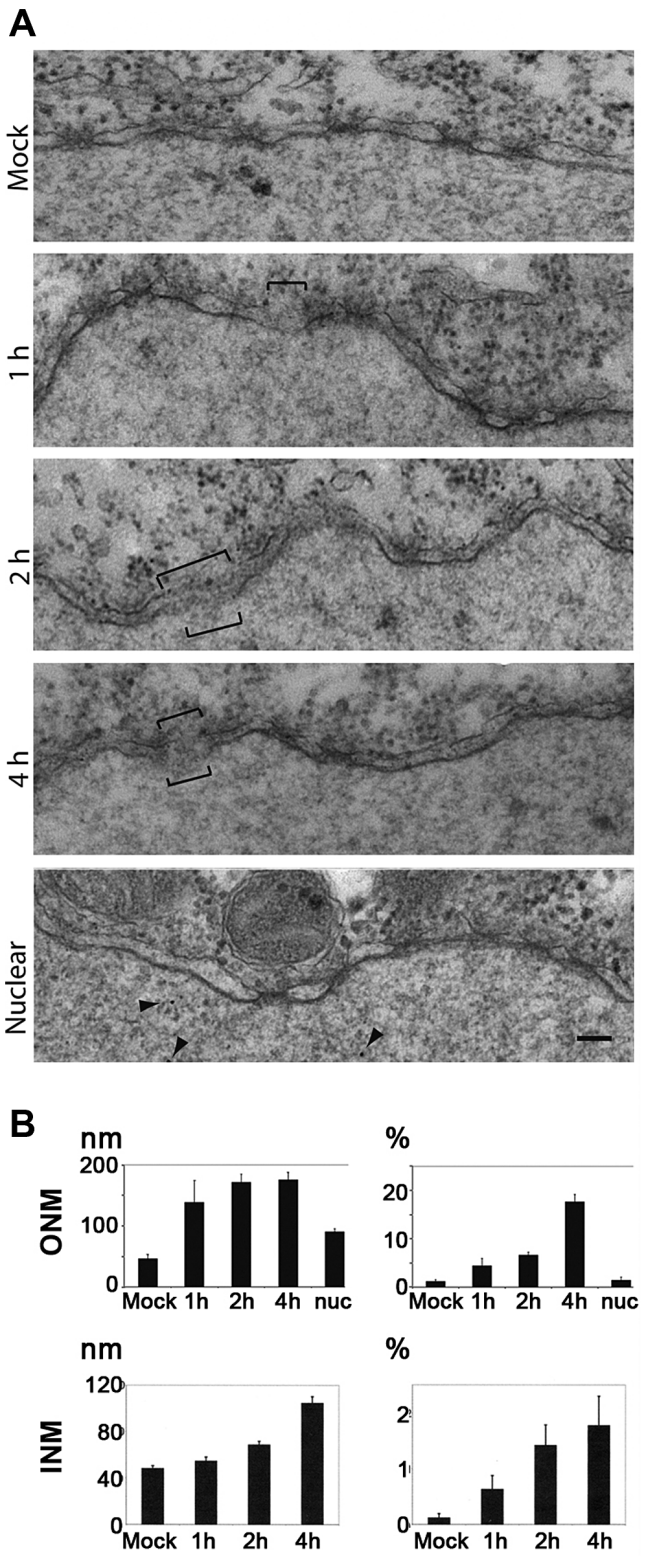
H1 causes nuclear envelope breaks in *Xenopus laevis* oocytes after microinjection into the cytoplasm. **A.** Electron microscopy of the nuclear membrane after microinjection of 2.13×10^5^ pfu./ml, or 2.17×10^8^ genomes/ml H1 into the cytoplasm of *Xenopus laevis* oocytes. The oocytes were fixed after 1, 2, 4 h prior to preparation of the nuclei and staining. Membrane breaks are indicated by brackets, the nucleus is on the bottom of each panel. MOCK: Tris EDTA, pH 7.8 injection and incubation for 4 h. Middle panels: injection of H1 with the indicated incubation time. Nuclear: nuclear microinjection (4 h RT). Bar = 100 nm. **B.** Quantification from 30 electron micrographs derived from microinjection into three oocytes per condition. Mock: mock-injected control. Nuc: nuclear microinjection after 4 h. Left panels: the length of the breaks. Right panels: proportion of degraded nuclear envelope. Nuc: nuclear microinjection. In summary these data show that PV-mediated NEBD leads to disruption of inner and outer nuclear membrane in *Xenopus laevis* oocytes, supporting that PV-mediated NEBD is a evolutionary well conserved process. The breaks in the membrane indicate that Ca^++^ leaks out of the lumen between the two membranes. The observation that ONM breaks occur with higher frequency than observed for the INM implies that membrane disintegration starts at the ONM.

H1 and acidified AAV2 bound at least to Nup358, which localises on the cytosolic face of the NPC, to Nup153 and probably Nup214, which both localize on both sides of the NPC [40], and to Nup62, which is part of the hydrophobic mesh filling nuclear pore [41]. Nup62 was not expected to be involved in NEBD as anti p62-antibodies do not block nuclear accumulation of AAV2 DNA or uptake of fluorescently labelled AAV2 into nuclei *in vitro* [42]. The external localization of Nups358 and 153 let us expect that VP1u causes local NEBD on the ONM first, followed by destruction of the INM. To test this idea we injected H1 into *Xenopus laevis* oocytes and analyzed local NEBD by EM. Figure 7A shows that PV H1-induced local membrane disruptions, which were not observed in buffer (MOCK)-injected oocytes. This observation is similar to the fenestration of the ONM recently observed in oocytes after microinjection of the minute virus of mice [27]. Number and size of the membrane breaks increased with the incubation time but their maximal size of ∼190 nm remained similar (Fig. 7B). We hypothesize that the difference between cell lines, which show larger breaks and the oocytes with restricted fenestrations by differences between somatic and germinal cells but we also considered that the nuclei of *Xenopus laevis* differ in their stability due to massive amounts of actin, which becomes exported upon fertilization [43].

Despite of differences in germinal vesicle breakdown and NEBD both systems are identical in terms of their nuclear pores and nuclear import [44]. We thus used the *Xenopus laevis* oocytes for analyzing the effect of nuclear H1 microinjection. Figure 7B showed few defects of the NE being in the same range than in MOCK-cytoplasmic injected oocytes. This observation is in agreement with infection during which progeny capsids accumulate inside the nucleus without nuclear disintegration.

INM disruption was only observed at those sites where the ONM was disrupted. INM breaks were however much rarer than ONM breaks suggesting that the ONM occurred before the INM was disrupted. The occurrence of breaks in the oocytes took much longer than in permeabilized cells, which could be caused by/reflect the low temperature at which the oocytes have to be incubated.

### Enzymatic activities are needed for NEBD

In addition to ONM and INM disruption, chromatin escape also requires lamina disintegration [45]. As shown in Supporting Information, figure S1A, Tx-100 mediated permeabilization of the nuclear membrane is not sufficient to cause even the release of a ∼100 kDa cargo. We thus analyzed the role of cellular enzymes essential for disassembly of the lamin network in mitosis and apoptosis. We inhibited PKC by the broad inhibitor H89 [46] and cdk-1/2 by roscovitine [47]. We used caspase-3 inhibition by zDEVD-fmk (not shown) and zVAD-fmk [48] as a control since it was shown that it is essential for nuclear disintegration by MVM infection [26] where it was demonstrated to be responsible for lamin degradation. The inhibitors we used blocked purified PKC, cdk-1/2 and caspase-3 specifically with regard to the other enzymes (Supporting Information, Fig. S6).

**Figure 8:**
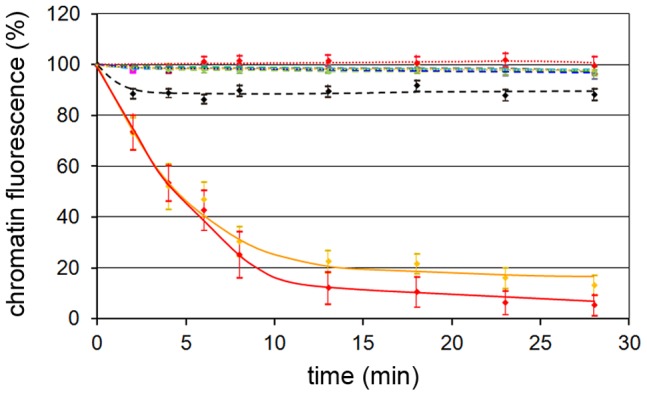
H1-mediated NEBD leads to simultaneous escape of a 100 kDa cargo and chromatin, which depends upon enzymes needed for mitosis. Nuclei of permeabilized cells preloaded with M9-Alexa647-BSA (M9) prior to addition of 300 H1 per permeabilized HeLa cell together with inhibitors. The graphs show quantifications of DAPI and Alexa647 fluorescences with the mean values and CI 95% (bars). Blue dotted line: DAPI with buffer only, no inhibitor (n = 39), pink dotted line: M9, buffer, no inhibitor (n = 39), orange line: DAPI, H1, no inhibitor (n = 21), red line: M9, H1, no inhibitor (n = 21), black line: DAPI, H1, H89 (n = 33), brown blue dashed line: M9, H1, H89 (n = 33), blue dashed line: DAPI, H1, roscovitine (n = 19), grey dashed line: M9, H1, roscovitine (n = 19), cyan dashed line: DAPI, H1, zVAD-fmk (n = 23), grey: M9, H1, zVAD-fmk (n = 23). Please note that some lines overlap. The figure shows that the escape of chromatin and the 100 kDa cargo cannot be separated despite of their different MW, indicating a catastrophic-like event during which the entire NE disintegrates. As all inhibitors entirely inhibited the loss of both cargos the results further indicate that PKC, cdks and caspase-3 are essential for NEBD.

Figure 8 shows that the caspase-inhibition in fact blocked the egress of a 100 kDa protein conjugate (M9-BSA), which was imported prior to H1 addition but also chromatin. As the specificity of caspase inhibitors is somewhat limited we further confirmed caspase-3 requirement using UV irradiated cells. Irradiation caused strong caspase-3 activation (Supporting Information, Fig. S7A), which significantly accelerated PV-mediated NEBD (Supporting Information, Fig. S7B).

PKC inhibition blocked the release of chromatin as well as the escape of the 100 kDa cargo. The involvement of PKC show thus some homology to the egress of CMV, which causes local PKC-dependent disassembly of the nuclear lamina [29]. In the non-inhibited control cells both M9-BSA and chromatin escape appeared at the same time implying that after onset of fenestration nuclear disintegration proceeds rapidly. This finding further supports the homology with NEBD during mitosis during which such rapid progression was described [5]. Furthermore we observed that cdk-inhibition blocked NEBD as PKC inhibition did, supporting the similarity of PV-mediated NEBD with mitosis in which cdk-1/2 are essential throughout pro-, meta- and anaphase. The observation that cdks and PKC activities were required implies that nuclear disintegration needed a coordinated activity of both enzyme families and that the activity of one could not rescue the missing activity of the other.

### PKC, cdk and caspase-3 are activated in a coordinated manner

**Figure 9:**
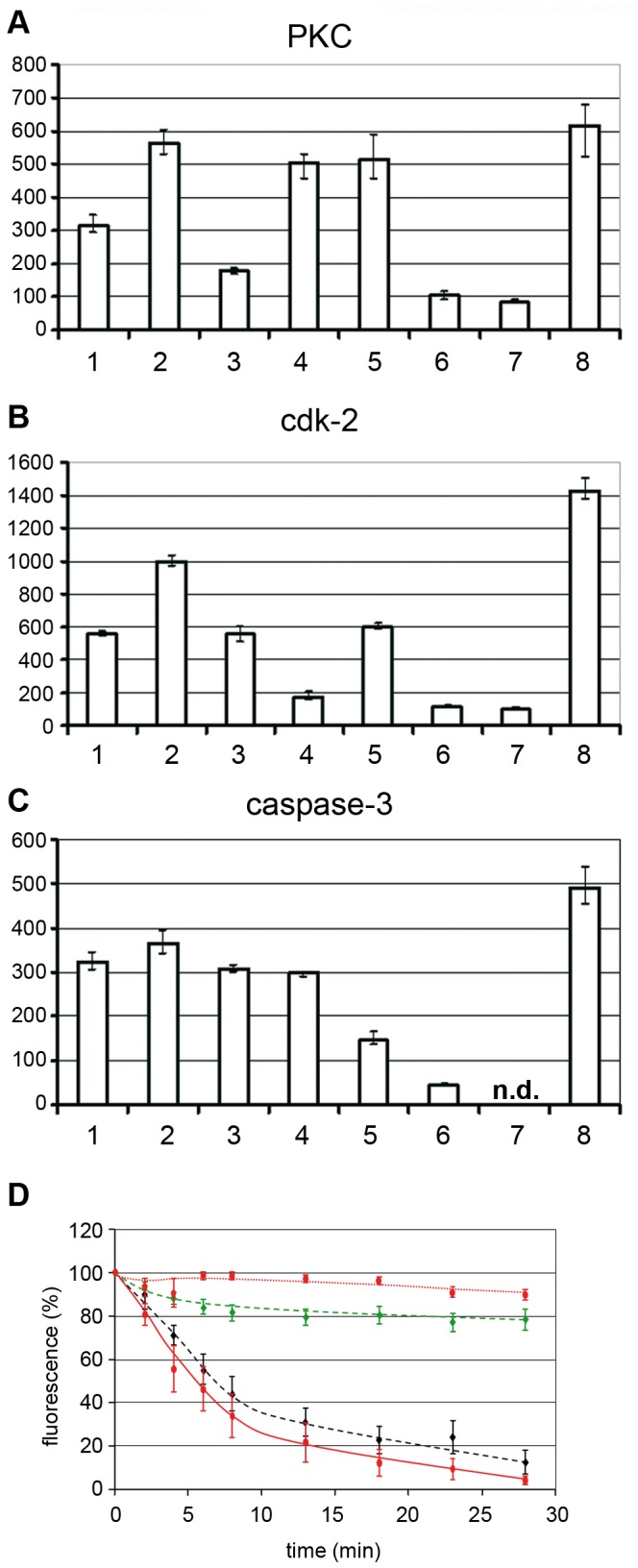
Cellular PKC and cdk-2 become activated by H1 and PKCα but not PKCβ is required for NEBD. **A, B, C:** Activation of PKC, cdk-2 and caspase-3 in permeabilized HeLa cells by H1. Y-axis: activity in arbitrary units. The tested activity is indicated on top of each panel. The columns show the mean values of three independent experiments. The variation bars show the range between the highest and the lowest value. 1. permeabilized cells. 2. permeabilized cells+H1. 3. permeabilized cells+H1+H89. 4. permeabilized cells+H1+Roscovitine. 5. permeabilized cells+H1+zVAD-fmk. 6. H1 without cells. 7. permeabilized cells+H1+thapsigargin. 8. non-permeabilized cells. n.d. not determined. The panels show that permeabilization decrease the activity of all three enzymes and that H1 activates the activities of PKC and cdk-2, while an effect on caspase-3 is doubtful. Inhibition of PKC also reduced activity of cdk2 but not of caspase_3 despite of its inhibitor specificity shown in the supporting information. Thapsigargin pre-treatment, leading to Ca^++^ depletion inhibits PKC and cdk2 implying that a Ca^++^-dependent PKC is involved. **D.** PV H1-mediated NEBD is inhibited by PKCα but not PKCβ. Quantification of PI-stained chromatin of permeabilized HeLa cells to which 300 H1 per permeabilized cell were added. The bars depict 95% CI. Red, dotted line: buffer (n = 28); red line: H1 (n = 14); green dashed line: H1 using PKCα-inhibited cells (n = 19); black dashed line: H1 using PKCβ-inhibited cells. Collectively, the data show that Ca^++^-dependent PKCα is required for NEBD, which is consistent with the PV-mediated activation of a Ca^++^-dependent PKC. PKCα subsequently activated cdk-2, which was also shown essential for NEBD. Caspase-3 was not significantly activated by PV but its activity was however essential for NEBD.

We next asked if there is a coordinated activation of the enzymes. We permeabilized cells, added H1 in the presence of the different inhibitors and analyzed the activity of i) PKC (α,β,γ,δ,ε,μ,θ,ζ), ii) cdk-2 and iii) caspase-3 in the lysates (Fig. 9A, B, C). We have chosen to analyze cdk-2 as it is needed during earlier steps of mitosis as G1/S phase transition. Its enzymatic activity could have been essential for NEBD as cdk-2 activates cdk-1 [49]. Cdk-1 in turn is required for lamin hyper phosphorylation, needed for lamin depolymerisation [50].

Permeabilization without H1 resulted in decreased activities of all enzymes to ∼50% due to the loss of the soluble cytoplasmic fractions (Fig. 9A, B, C). Adding H1 to the permeabilized cells doubled PKC and cdk-2 activities (Fig. 9A, B), while caspase-3 activity was not significantly altered (Fig. 9C). Consistently, inhibition of PKC by H89 or of cdk-2 by roscovitine did not affect caspase-3 activity. In contrast H89 inhibited not only PKC (Fig. 9A) but also cdk-2 (Fig. 9B) implying that PKC was activated first, which then activated cdk-2. This finding shows that there is also an indirect stimulating effect of PV on cdk-2, which might counteract the inhibitory function of PV as it was shown by biochemical assays using purified cdk-2 and AAV2 and 8 [51]. Roscovitine in turn did not change PKC activity (Fig. 9A) supporting that PKC activation is independent upon cdk-2 activity but is required for cdk-2 activation. Cdk-2 became as well inhibited by caspase-3 inhibition (Fig. 9B), which is in agreement with observations of others who showed a caspase-3 dependent activation of cdk-2 [52]. PKC activity was however not affected by caspase-3 inhibition (Fig. 9A), suggesting that no apoptosis-related PKCδ cleavage occurred.

As the detection system for PKC comprised the detection of untypical Ca^++^-independent PKCs we asked for the impact of Ca^++^, assuming that Ca^++^ could have escaped from the lumen between ONM and INM upon membrane disruption. We pretreated HeLa cells with thapsigargin, which depletes Ca^++^ by inhibiting the endoplasmic reticulum Ca^++^ ATPase [53]. Figure 9A shows that Ca^++^ depletion restricted PKC activity in the permeabilized cells to the same extent as H89, supporting that the Ca^++^-independent proapoptotic PKCδ was not required. The finding is in agreement with observations of others showing that calcium chelator treatment prevents mitotic NEBD in sea urchins [54]. Thapsigargin-treatment also inhibited cdk-2 (Fig. 9B), which is in accordance with the observation that PKC has to become activated for subsequent activation of cdk-2.

H89 is a relatively broad inhibitor of PKCs but the Ca^++^ dependence of the activations suggested that the PKC isoform involved in PV-mediated NEBD is also Ca^++^ dependent, thus involving PKCα, β and γ. PKCγ is neuron-specific, while PKCβ is involved in lamin phosphorylation [55]. PKCα also causes lamin phosphorylation [56] and its inhibition results in cell cycle arrest [57]. In order to decipher their function in PV-mediated NEBD we added H1 to permeabilized cells, which have been pretreated for 1 h with either LY333531 (an established inhibitor of PKCβ) or with a myristylated pseudo substrate peptide inhibiting PKCα. This inhibitor is considered to be highly specific as it acts as regulatory domain of PKCα suppressing activity of the catalytic domain [58]. Figure 9D showed that inhibition of PKCα strongly inhibited PV-mediated NEBD but that inhibition of PKCβ had only a marginal effect. In view of the results shown before we conclude that three enzymes which are either essential (PKCα and cdk-1/2) or discussed to be involved (caspase-3) in mitosis were also indispensable for PV-mediated NEBD. Further PKC and cdk-2 became activated in a Ca^++^-dependent manner; the activation of the latter – although Ca^++^-independent - as a consequence of PKC activation. Consistently, the Ca^++^-dependent PKCα but not PKCβ was identified as essential for PV-mediated NEBD.

### Nuclear permeabilization upon microinjection of H1 and Ca^++^

**Figure 10:**
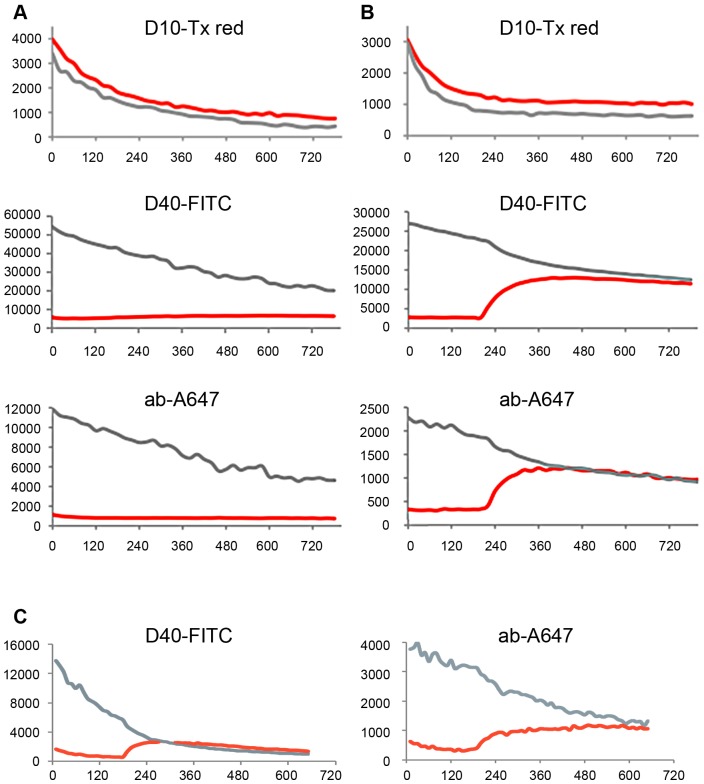
Quantification of fluorescence intensity of the markers microinjected with or without H1 into U2OS cells. Quantifications of the fluorescences presented as videos in the supporting information. The microinjected markers are indicated on top of each panel (D10-Tx: Dextran 10-Texas Red labelled, D40-FITC: Dextran 40, FITC-labelled, ab-A647: unrelated IgG, Alexa.647-labelled). The grey lines show the cytoplasmic, the red line the nuclear fluorescence. The y-axis depicts the intensity given in arbitrary units, the x-axis the time after microinjection in seconds. **A.** Microinjection of buffer with marker proteins. D10-Tx equilibrated between cytoplasm and nucleus directly after microinjection while D40-FITC and ab-A647 stayed excluded from nucleus. **B.** Microinjection of H1 with marker proteins. D40-Tx and ab-A647 entered the nucleus simultaneously 240 seconds after microinjection and reached the equilibrium. **C.** Microinjection of Ca^++^ with marker proteins. D40-Tx and ab-A647 entered the nucleus simultaneously 200 seconds after microinjection also reaching the equilibrium. In summery the panels show that both H1 and Ca^++^ triggered sudden NEBD approx. 2–3 min after microinjection. Dextran 40 and the antibodies entered the nucleus at the same time indicating a catastrophic-like destruction of the barrier as it was also seen for nuclear escape of 100 kDa cargos and chromatin in permeabilized cells.

In order to link our in vivo infection data with the results obtained in permeabilized cells we used microinjection of H1. By time lapse microscopy we visualized the kinetics and extend of NEBD. For this approach we first co-injected differential fluorescently labelled marker molecules of increasing size alone or together with H1 into the cytoplasm of U2OS cells. Following injection in the absence of H1 a 10 kDa marker molecule (5 nm in diameter) equilibrated rapidly between nucleus and cytoplasm because its small size allows diffusion across the NPC barrier (Fig. 10A). A 40 kDa marker (10 nm in diameter) and a 150 kDa marker (IgG antibodies; 15 nm in diameter) stayed cytoplasmic, which is in agreement with the size exclusion limit of the NPC (Figure 10A and supplemental movie S1). Both 40 kDa and 150 kDa marker rapidly equilibrated in the cytoplasm. Given the linear relation between diameter and diffusion this observation indicates that also H1 (26 nm diameter) can reach the NE within less than a minute.

Upon co-injection of ∼100 H1 particles we observed NEBD within minutes after injection indicated by the simultaneous entry of the 40 and 150 kDa marker (Figure 10B). Considering that 100 kDa markers were retained in the nuclei devoid of their nuclear membrane (Supporting Information, Fig. S1A) we conclude that the entry of at least the 150 kDa marker upon microinjection indicate lamin depolymerization as it occurred in permeabilized cells (Fig. 3A, B). The increase in NE permeability occurred at once without specific localization resembling a catastrophic event (supplemental movie S2), which corresponds to the isochronic escape of the 100 kDa cargo and the chromatin in permeabilized cells (Fig. 8). The sudden appearance of NEBD is a characteristic of cdk-1 activation (in the complex with cyclin A2) upon mitosis [59]. The onset of NE permeability was observed on average after 290 sec (95% CI from 128 to 395 sec., 31 cells) and both markers were in equilibrium in cytoplasm and in nucleus supporting the idea of a massive increase of permeability. In fact this timescale was thus similar to that observed in permeabilized cells. Entry was simultaneous for the 40 kDa and 150 kDa markers, which is in accordance to the simultaneous egress of the 100 kDa protein and chromatin in permeabilized cells (Figure 8).

Considering that only 11% of the infected cells showed NEBD while the nuclei of all permeabilized or microinjected cells were disintegrated we thus conclude that limited endosomal escape of PV accounted for the restricted number in infection as it was reported previously [60].

The importance of Ca^++^ shown by Thapsigargin-treatment (Fig. 9) and the need of the Ca^++^-dependent PKCα let us ask if we could trigger NEBD directly by Ca^++^ circumventing the Ca^++^ release from the nuclear envelope by PV. We microinjected Ca^++^ to a final concentration of 7 mM, which is the concentration between the inner and out leaflet of the NE [61]. The intracellular concentration upon microinjection was determined by comparing the fluorescence of the co-injected fluorescent markers (40 kDa Dextran and IgG antibodies) with a standard dilution series. Measuring the permeability by time-lapse microscopy as described before we observed a sudden onset of nuclear influx of both markers 200 sec after microinjection (Fig. 10C and supplemental movie S3). This finding is in agreement with observations of others showing that Ca^++^ is sufficient for driving mitosis in early mouse embryos [62]. The observation that NEBD occurred around the same time than upon PV microinjection supports that PV caused Ca^++^ release, which is the initial cellular trigger for starting the cascade of PKC and cdk activation leading to NEBD.

### Summary

**Figure 11:**
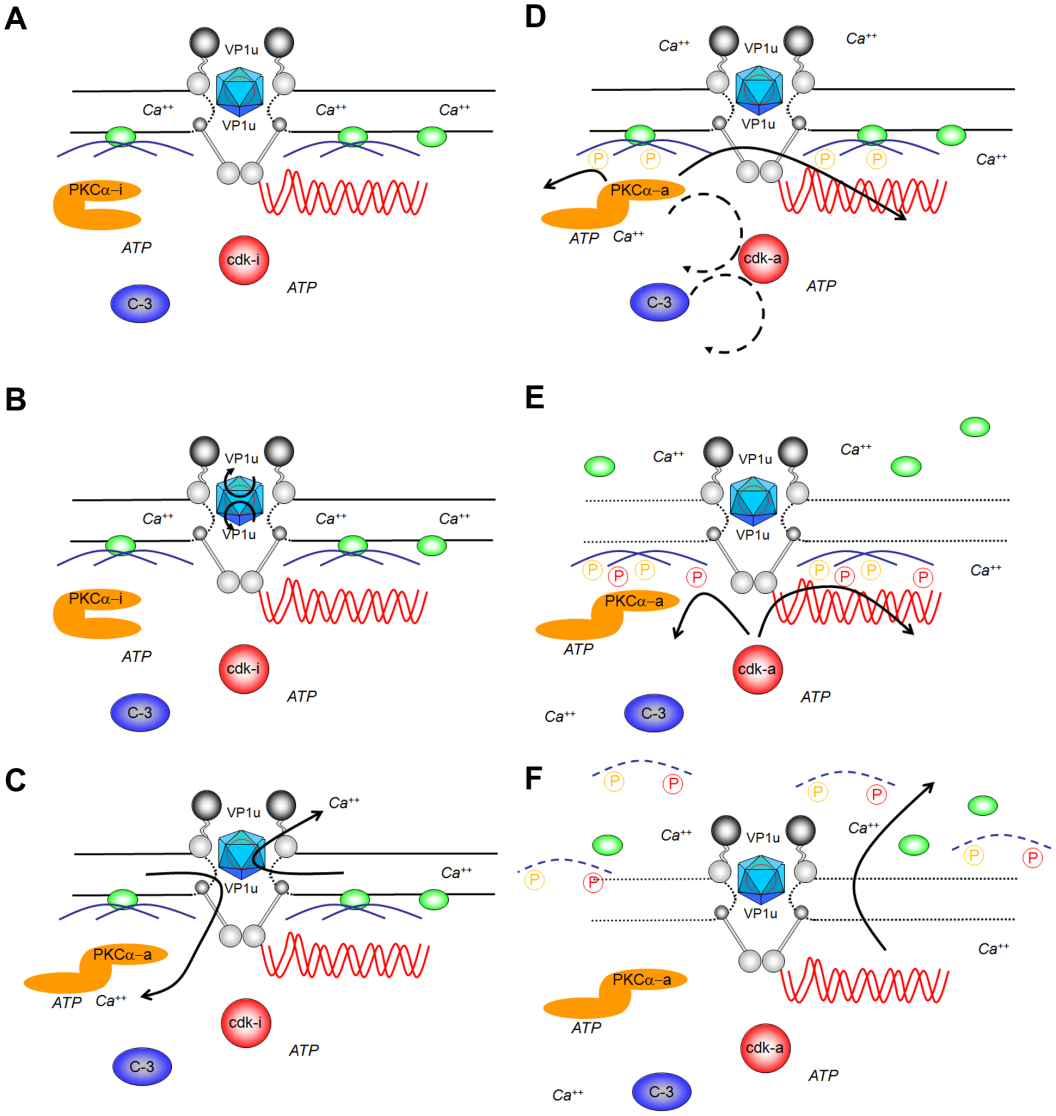
Schematic presentation of the parvoviral interaction with the nuclear envelope. **A.** Upon arrival at the nuclear pores parvoviral capsids (blue icosahedra) interact directly with at least three Nup (Nup358: dark grey; Nup153: light grey, Nup62: middle grey). **B.** This interaction causes exposure of VP1u on the surface of the virus. **C.** VP1u exposure allows permeabilization of the nuclear membrane, which is indicated by the dotted line of the NE (black lines). The permeabilization causes efflux of Ca^++^ indicated by the arrows, causing elevated local Ca^++^ in the nuclear periphery. **D.** Ca^++^ activated PKCα causing exposure of the catalytic domain (orange; PKCα-i: inactive; PKCα-a: active), which than phosphorylates lamins (blue; PKCα phosphorylation indicated by orange P). Further PKCα inactivates indirectly cdk-2, subsequently activating cdk-1 (indirect activations indicated by a dotted circle; cdks in red, cdk-i: inactive, cdk-a: active). Further activation is mediated by caspase-3, likely in an indirect manner. **E.** Active cdk1 hyper phosphorylates lamins, shown by red P. Hyper phosphorylation subsequently causes lamin depolymerisation. On combination with the spread of membrane disintegration the LBR (green) dissociates from the NE. **F.** After significant membrane disintegration even larger structures as chromatin (red waved lines) can escape the nucleus.

In summary our data give evidence for a unique virus-mediated pathway that causes NEBD (see scheme Fig. 11). NEBD was observed for different PV and in cells ranging from human to *Xenopus laevis* implying an evolutionary well conserved phenomenon. Our model of PV host-interaction starts with the release of the viruses from the microtubules in the nuclear periphery, followed by (a) attachment to the NPC directly, which (b) subsequently causes exposure of VP1u. A yet undefined domain of VP1u then (c) permeabilizes the nuclear membranes leading to Ca^++^ efflux. (d) Ca^++^ activates nuclear PKCα, which phosphorylates lamins [56] and activates cdk-2, which becomes further activated by caspase-3 as described recently [63]. Activated cdk-2 – a key element of entry into mitosis - possibly leads to cdk-1 activation and (e) lamin A/C hyper phosphorylation [64]. (f) Lamin (hyper) phosphorylation than leads to lamin depolymerisation allowing entry and exit of large cargos from the nucleus by diffusion. At least in somatic cells the activation cascade must spread within the nucleus explaining why nuclear permeability increased suddenly, which is also a characteristic of mitosis.

The PV-mediated NEBD apparently by-pass the early mechanisms of NEBD during mitosis entry, explaining the observed differences to mitosis. In consequence we have not observed chromatin condensation taking place during prophase. Although we have not investigated tubulin polymerization causing permeabilizing the nuclear membrane [5] and leading to Ca^++^ efflux, we assume that the pool of tubulin at least in permeabilized cells does not allow their formation. Instead PV cause Ca^++^ release directly from the space between INM and ONM as it occurs directly before NEBD [6]. The evasion of PV thus allows direct entry into the mitotic pathways at a later stage.

Despite of significant differences to NEBD in mitosis we conclude that PV use the mitotic pathway for causing nuclear envelope disintegration. The homologies comprise not only the need of PKCα and cdk-1/2, which are implied in lamin depolymerization by phosphorylation [8] and cell cycle progression [65], [66] but also the sudden onset and rapid progression [5]. The need of caspase-3 for PV-mediated NEBD seems surprising in this context but its implication in mitosis is controversial [9]–[11]. Evidently there are striking differences to NEBD in apoptosis not only in that it takes muck longer [32] but also as apoptosis requires PKCδ [12] and not PKCα. Furthermore, we never observed DNA fragmentation or the characteristic DNA patches in the nuclear periphery.

Assuming that PV use the mitotic pathways it was most surprising that virus-mediated NEBD was driven solely by nuclear factors. However although our study does not entirely explain the molecular mechanisms and activations for NEBD it reveals that pathways mediating NEBD can be uncoupled from the various checkpoints of mitosis. We thus assume that PV provide a new entry point to unravel the functions of various proteins upon the different stages of mitosis.

## Materials and Methods

### Cell lines and virus

NBK-324, U2OS and HeLa cells were grown in DMEM/5% FCS at 37°C. EYFP lamin B receptor NRK cells were grown in DMEM/8% FCS. Viral stocks were generated by infecting NBK-324 cells with 0.1 pfu viruses per cell and harvested after 2.5 days. Virus stocks were extracted from infected cells by cell lyses in 50 mM Tris-HCl, 0.5 mM EDTA and 5 cycles of freezing and thawing. Viruses were purified by iodixanol step gradient centrifugation [67].To analyze the effect of acidification on parvoviruses, the viruses were incubated for 10 min in sodium acetate buffer pH 5.2, followed by neutralisation with 0.5 M Tris base.

### Infection of HeLa cells by parvovirus H1

HeLa cells were seeded on collagenized cover slips and grown overnight prior to infection with 1000 H1/cell. Four h post infection the cells were washed with DMEM and fixed with 3% paraformaldehyde (Merck)/PBS at 4°C for 2 h. Cells were washed twice in PBS, permeabilized with 0.1% Triton X-100/PBS for 10 min at RT, washed 3× with PBS and incubated in blocking solution (0.5% BSA/5% goat serum/PBS) for 15 min at 37°C. Primary antibodies were diluted in blocking solution (rabbit polyclonal anti VP1/VP2 1∶100; mAb414 (Hiss Diagnostic) 1∶500) and added to the cells for 90 min at 37°C in a humidified chamber. Cells were washed in 4× in PBS, before 1∶200 diluted secondary antibodies (FITC-conjugated goat anti-rabbit 1∶200, Cy5-conjugated goat anti-mouse antibody (Jackson Immuno Research) with 0.2 µg/ml propidium iodide was added for 45 min at 37°C. After 4 washes with PBS, the cover slips were mounted on glass slides using 50 mg/ml DABCO/Moviol.

### Microscopy and image analysis

Real time microscopy was performed using a Leica SP-5 confocal microscope equipped with 3 internal PMT and 1 PMT trans, using a HCX Plan Apo CS 20X multi-immersion NA 0.70 lens at 37°C in transport buffer (200 mM Hepes pH 7.3, 20 mM magnesium acetate, 100 mM potassium acetate, 50 mM sodium acetate, 10 mM EGTA) or life-cell imaging media (Invitogen). Images were acquired using the standard setting of the microscope and a pinhole size of 1.0 and the LEICA acquisition software LAS AF. In the experiments with permeabilized cells images were taken at the indicated time points and quantification of the stained nuclei was done using Image J followed by analysis using excel data sheets.

In microinjection experiments of somatic cells the images were taken at a frame rate of 1 frame per 15 seconds for a total of 40 frames and mounted in image J. Correction for photo bleaching was performed using Image J with a plugin described elsewhere (http://fiji.sc/wiki/index.php/Bleach_Correction).

Microscopy of fixed cells was done at RT using an HCX Plan Apo CS 40X oil NA 1.25 lens. 3D reconstruction images were captured by confocal microscopy and reconstitute by Imaris software.

### H1 on unpermeabilized HeLa cells

HeLa cells were grown on collagenized cover slips overnight at 37°C. Cells were washed and 300 H1 viruses was added for 15 min at 37°C in medium followed by washing. Cells were stained with propidium iodide for 5 min at RT and mounted.

### Parvovirus-mediated nuclear degradation in digitonin-permeabilized cells

1×10^5^ cells were grown on collagenized 12 mm cover slips, washed twice with serum free media, before serum-free medium/20 µg/ml digitonin/1 µg/ml propidium iodide was added. After incubation for 5 min at 37°C cells were washed with ice-cold transport buffer. The cover slips were placed in a heating device and the propidium iodide stain was used to focus the samples. The buffer was than replaced by 37°C pre-warmed virus in transport buffer (20 mM Hepes [pH 7.3], 2 mM Mg-acetate, 110 mM K-acetate, 5 mM Na-acetate, 1 mM EGTA)/2 mM DTT (100 µl) or by new pre-warmed transport buffer/2 mM DTT (negative controls). Modifications are indicated in the individual experiments. When cargos were imported into the nuclei or when the nuclei were preincubated with hepatitis B virus capsids prior to addition of parvoviruses the washed, permeabilized cells were subjected to rabbit reticulocyte lysate (21 mg/ml, Promega) in transport buffer/2 mM DTT/20 U/ml creatine phosphokinase/5 mM creatine phosphate, containing 150 µg/µl M9-Alexa 647-BSA, or 150 µg/µl NLS-Alexa 594-BSA or 1200 ng capsids for 15 min at 37°C. After 3 washes with transport buffer, parvoviruses were added as described.

### Microinjection into *Xenopus laevis* oocytes and somatic cells

Oocytes were microinjected and prepared for thin sectioning EM as previously described [27]. Oocytes were injected with about 100 nl of purified H1 (2.13×10^9^ pfu./ml, or 2.17×10^12^ genomes/ml) in the cytoplasm at the transitional zone between the animal and vegetal poles. As control experiments, oocytes were mock injected with 100 nl Tris-EDTA buffer (TE: 50 mM Tris, 0.5 mM EDTA, pH 8.7). Oocytes were then incubated at RT in modified Barth's saline buffer (MBS: 88 mM NaCl, 1 mM KCl, 0.82 mM MgSO_4_, 0.33 mM Ca(NO_3_)_2_, 0.41 mM CaCl_2_, 10 mM HEPES, pH 7.5).

After microinjection and incubation at RT, oocytes were fixed o.n. at 4°C with 2% glutaraldehyde in MBS. Oocytes were washed with MBS and their animal poles were dissected and fixed with 2% glutaraldehyde in low-salt buffer (LSB: 1 mM KCl, 0.5 mM MgCl_2_, 10 mM Hepes, pH 7.5) for 1 h at RT. Dissected oocytes were washed with LSB, embedded in 2% low melting agarose and post-fixed with 1% OsO_4_. Fixed oocytes were sequentially dehydrated in ethanol and embedded in Epon 812 (Fluka) as described elsewhere [68]. Following embedding, 50-nm thin sections through the nuclear envelope (NE) were cut and placed on phalloidin/carbon coated copper EM grids, stained with 2% uranyl acetate for 30 min and 2% lead citrate for 5 min, and viewed with a Hitachi-7600 transmission electron microscope.

Nuclear envelope disruption was quantified by measuring the length of outer nuclear membrane (ONM) and inner nuclear membrane (INM) disruptions from EM cross-sections of NE using Carnoy image analysis software (Biovolution). Bar graphs represent the average length of the ONM breaks, or the average proportion of NE damage calculated as the length of the ONM breaks divided by the total length of the ONM from electron micrographs.

Microinjections into U2OS cells were performed using an Eppendorff FemtoJet microinjection device coupled to a LEICA SP5 confocal microscope. Injection solutions contained Texas Red coupled Dextran (10 kDa, 2 mg/ml final concentration), FITC coupled Dextran (40 kDa, 1 mg/ml final concentration) and Alexa647 coupled secondary mouse antibodies (150 kDa, 0.4 mg/ml final concentration) all diluted in transport buffer.

### Isolation of nucleoporins

Seventy-five % dense HeLa cells from ten 16 cm dishes were treated with 4.2 mM Cytochalasin B/DMEM for 30 min at 37°C. Cells were trypsinized and resuspended in 10 ml PBS. Cells were sedimented at 200× g for 10 min at 4°C. Washing and centrifugation steps were repeated 3 times. The pellet was resuspended in 5 ml nuclei buffer (10 mM PIPES (pH 7.4), 10 mM KCl, 2 mM MgCl_2_, 1 mM DTT) and the cells were sedimented at 200× g for 10 min at 4°C. The cells were resuspended in 10 volumes nuclei buffer/10 µM Cytochalasin B, incubate for 30 min on ice and homogenized by 30 strokes on ice. The sample was loaded on 4 volumes of 30% (w/w) sucrose/nuclei buffer and centrifuged at 800× g and 4°C for 10 min. The pellet was resuspended in 500 µl nuclei buffer and the centrifugation step was repeated. The nuclei-containing sediment was washed with 3 ml of nuclei buffer and the nucleoporins were isolated according to [69]. The nucleoporins were quantified by Bradford assay (Biorad, Germany).

### Western blots

#### Tubulin blot

HeLa cells (permeabilized and non-permeabilized) were sedimented at 1000× g force for 5 min at 4°C and lysed in SDS lysis buffer (Invitrogen, Heidelberg, Germany) prior to loading onto .10–20% Tris-Acetate SDS PAGE (Invitrogen). After transfer of the proteins to a PVDF membrane, the membrane was blocked in blocking buffer (5% skim milk in PBS) for 1 h at RT. Primary antibody TAT-1 specific to α- tubulin (1∶50) was added in PBS + 0.2% Tween 20 + 0.5 M NaCl + 0.02% NaN_3_ o.n. at 4°C. The membrane was washed 3 times with 1 M NaCl in H_2_O for 10 min, then incubated with peroxidase-labelled anti mouse antibody for 1 h at RT followed by 4 washes with 0.2% Tween 20/PBS for 10 min. The tubulin was visualized using an ECL detection kit (Perkin Elmer) on hyper films MP (X-ray films, Amersham Biosciences, Germany).

#### Nucleoporin blot

6.7×10^6^ sheep anti rabbit-coated biomagnetic beads (Dynal) were washed 2 times with 0.1% BSA/PBS prior to addition of anti VP1/2 antibodies. After incubation o.n. at 4°C on a rotating wheel the beads were washed 4 times with 0.1% BSA/PBS. The beads were resuspended in 1000 µl transport buffer containing 150 ng parvoviruses and 35 µg of nucleoporins and incubated o.n. at 4°C on a rotating wheel. Samples were washed 3× with 0.1% BSA in PBS then 1× with 0.1% NP-40 in PBS, transfer the samples in new cups, later washed the samples 4 times with PBS. The samples were loaded on a 3–8% Tris Acetate SDS PAGE, blotted onto a PDVF membrane, which was blocked by 5% skim milk in PBS for 1 h at RT. MAb414, which reacts with FG repeat-containing nucleoporins, was added in a dilution of 1∶3000 in 5% skim milk in PBS for 3 h at RT. The subsequent procedure is described above.

### Activity tests

#### PLA_2_

150 ng parvoviruses were pre-incubated in presence or absence of nucleoporins (30 µg/sample) in transport buffer and 1 mM CaCl_2_ for 2 hrs at 4°C prior to addition to a c-PLA_2_ assay kit (Cayman, USA), which monitors all kind of PLA_2_ activities.

Protein kinase C, cyclin-dependent kinase, caspase-3. 2.5×10^6^ HeLa cells were grown overnight at 37°C on 10 cm dishes before 10 µM H-89, 50 µM Roscovitine, 50 µM ZVAD-fmk or 1 µM Thapsigargin (Calbiochem) was added for 2 h at 37°C. Thereafter, cells were washed with serum free media and permeabilized with digitonin as described above. The permeabilized cells were harvested and washed three times at 4°C by sedimentation at 1,000× g for 5 min and resuspension. 300 parvoviruses were added to the 1.5×10^4^ permeabilized cells and the activity was measured after lyses by 3 freeze and thawing cycles by either PKC kit (Assay Designs, USA), or Apo-one homogeneous Caspase-3 kit (Promega), or CDK2/CycA Kinase assay kit (Cell Signalling, USA) according to the vendors protocol. The protein kinase assays use enzyme-specific peptides, which are bound by phosphorylation-specific antibodies. The caspase assay uses a fluorescence-labelled peptide, which becomes cleaved.

### Caspase-3 in living cells

HeLa cells were seeded on collagenized cover slips o.n. at 37°C. Cells were washed 2 times with serum free medium followed by UV irradiation for 30 sec (9,000 µW/cm^2^, λ = 312 nm). One hundred µl of Phiphilux (OncoImmunin) and 10 µl of foetal calf serum were added to the cells for 1 h at 37°C. Cells were washed with PBS and images were taken by confocal laser scanning microscope.

## Supporting Information

Figure S1 **Permeabilized HeLa cells do not contain significant amounts of soluble cytosolic proteins.** **A.** Nuclear import assay of karyophilic substrates, visualized by LSM. NPC: indirect fluorescence of the NE using mAb414, which binds to different proteins of the nuclear pore; M9: direct fluorescence of nuclear M9-Alexa647-BSA, NLS: direct fluorescence of nuclear NLS-Alexa594-BSA conjugate. First row: permeabilized cells+both karyophilic cargos (M9-Alexa647-BSA and NLS-Alexa594-BSA)+exogenous cytosolic extract. Second row: as before but in the absence of cytosol. Third row: permeabilized cells+exogenous cytosolic extract without cargos. Fourth row: import as in the first row, followed by treatment with Tx-100 to remove the membrane. The figure shows that no visible active nuclear import occurs in permeabilized HeLa cells after permeabilization and washing. Replacement of the cytosolic factors by cytosol reconstitutes the nuclear import capacity of the nuclei. Lower row: control reaction to test whether permeabilization of the nuclear membrane alone cause escape of M9-Alexa647-BSA from the nucleus. The nuclei of the digitonin-permeabilized HeLa cells were loaded by the karyophilic cargos in the presence of cytosolic extract as in the upper two rows, then washed and incubated with 0.5% Tx-100 at RT until the nuclei lost contact from the cover slip. The nuclei were sedimented onto collagenized cover slips, fixed and the NPC was stained by indirect immune fluorescence. The loss of NPC stain indicates that the Tx-100 treatment removed the NE including the integrated NPCs. Bar = 25 µm. **B.** Immune detection of α tubulin after SDS PAGE in unpermeabilized and Digitonin-permeabilized cells. The arrow indicates the migration of α tubulin (66 kDa). The treatment of the cells is indicated on top of the figure 1. Lysate of 2.5×10^6^ HeLa cells, 2. 1∶4, 3. 1∶16, 4. 1∶64, 5. 1∶ 256, 6. 1∶1024 dilutions. The blot shows that upon permeabilization and washing on ice – which depolymerizes microtubules - less than 2% of α tubulin remained in the cells. In summary the figure shows that permeabilized cells are practically free of soluble cytosolic proteins not allowing any active transport.
(TIF)

Figure S2 **H1-mediated NEBD is dose-dependent but independent upon contaminating factors.** Quantifications of the PI fluorescences in permeabilized HeLa cell nuclei with the mean values and the 95% confidence intervals (bars). X-axes: time in min; y axes: relative PI fluorescence. **A.** Dose-response curve of chromatin escape. Blue: 37.5 genome-containing H1 particles permeabilized cell (n = 12), pink: 150 H1 particles permeabilized cell (n = 9), red: 300 H1 particles permeabilized cell (n = 10), green: 600 H1 particles permeabilized cell (n = 6). **B.** Loss of chromatin is independent upon H1 preparation method. Red line: buffer only (n = 5), black: 300 iodixanol-purified H1 permeabilized HeLa cell (n = 8), green: 300 CsCl gradient-purified H1 permeabilized cell (n = 5), cyan: iodixanol MOCK-purification, in which uninfected cells were subjected to the purification protocol used for H1 (n = 7). Although NEBD was visible for both H1 preparations but not for the MOCK control the CsCl gradient preparation showed a slower kinetic. This is in agreement with a lower infectivity of PV upon this purification protocol (not shown). **C.** Silver stain of iodixanol gradient purified H1 after SDS PAGE. The MW of the marker proteins are shown on the right, the H1 proteins are indicated on the left of the gel. The silver stain exhibits the three structural proteins of H1, VP1, VP2 and VP3 with their characteristic MW. Three faint additional bands are visible with a MW of approximately 50 kDa. The Western blot confirms that these bands are reactive for the anti H1 antibody (not shown). The purity of the CsCl gradient purified capsids is shown elsewhere [1] showing exclusively VP1, VP2 and VP3. In summary the data show that PV-mediated NEBD is not caused by contaminating factors of the H1 preparation.
(TIF)

Figure S3 **H1 mediated NEBD is temperature and energy dependent.** **A.** Temperature-dependence of chromatin escape using 300 H1 per permeabilized HeLa cell. The graph depicts the mean values and the 95% confidence intervals (bars) as in Fig. S2. Black line: buffer only at 37°C (n = 7), red line: H1 at RT (n = 9), green line H1 at 37°C (n = 8). The graph shows that H1 at RT causes a 50% loss of PI fluorescence occurred after 30 min, while a 50% reduction at 37°C occurred after 5 min. **B.** Energy-dependence of H1-mediated chromatin escape. Red line: buffer only (n = 34), green line: buffer with hexokinase/glucose, which depletes ATP and GTP from the permeabilized cells (n = 32), blue line: hexokinase/glucose with 300 H1 per permeabilized cell (n = 27), black line: 300 H1 per permeabilized cell (n = 23). The two graphs show that H1-mediated NEBD is an energy- and temperature-dependent process, indicating the need of enzymatic activities.
(TIF)

Figure S4 **Analysis of the Nup preparation.** **A.** Silver stain after SDS PAGE. The MW of the marker is given on the left. 1: 250 ng, 2: 125 ng, 3: 62.5, 4: 31.3 ng of importin α and importin β each. M: MW marker, N: nine µg (total protein) of the Nup preparation. **B.** Western blot of the lysates from intact HeLa cells and of the Nup preparation after SDS PAGE using mAbs against importin α, importin β and an antibody against Nups 358, 214, 153 and 62 (mAb414). The MW of the marker is given on the left, The migration of the different proteins is indicated on the right. 1: 5×10^6^ cells, 2: 1×10^6^ cells, 3: 2×10^5^ cells, 4: 4×10^4^ cells, 5: 8×10^3^ cells. 6: 18 µg (total protein) of the Nup preparation, 7: 36 µg. Based on Nup62, which is an internal protein of the NPC, it can be concluded that 36 µg of the Nup preparation contain the Nups of 2×10^6^ cells. The importin α signal is however much weaker than in 8×10^3^ cells indicating a reduction of more than 500fold. Importin β was reduced at least 50fold. The relative strength of the Nup bands changed between intact cells and the Nup preparation indicating that the NPC were separated into isolated Nups.
(TIF)

Figure S5 **H1 does not degrade the plasma membrane of HeLa cells.** Wide field microscopy of HeLa cells. Left: phase contrast, right: PI fluorescence. H1: Three hundred H1, in a concentration corresponding to the conditions used in cells with permeabilized cells were added for 15 min at 37°C in medium mixed with PI. . ∅: control with PI/medium. As PI is cell impermeable the absence of significant stain indicates that the cells stayed intact and that H1 do not degrade membranes unspecifically. Bar = 10 µm.
(TIF)

Figure S6 **Specificity of inhibitors against PKC, cdk-2 and caspase-3.** **A.** Mixture of PKC isoforms (α,β,γ,δ,ε,μ,θ,ζ): 1. no inhibitor, 2. 10 µM H89, 3. 50 µM roscovitine, 4. 50 µM zVAD-fmk. Y-axis: relative activity in % of the non-inhibited control. The bars indicate the range of 3 independent assays **B.** Cdk-2. Columns as in A. The panels show that PKC and cdk-2 were inhibited to ∼20% by the corresponding inhibitor but not affected by the inhibitors of the other protein kinase. Thapsigargin showed a slight inhibition of 20%. Thapsigargin was removed prior to permeabilization and PV addition, excluding significant cross inhibition. In summary the figures show that there was no significant unspecific inhibition of the inhibitors regarding to the tested enzymes.
(TIF)

Figure S7 **Caspase-3 activation increases the speed of H1-mediated chromatin escape.** **A.** Phiphilux fluorescence of HeLa cells 1 h after irradiation with 9,000 µW/cm^2^ UV light (λ = 312 nm). Bar = 20 µm. The figure shows that UV light strongly induced caspase-3 activity mainly in the cytoplasm but also intranuclear. **B.** H1-mediated chromatin escape of permeabilized UV-irradiated cells by 300 H1 permeabilized cell. The graphs show quantifications of the PI fluorescences with the mean values and the 95% confidence intervals (bars). Red line: buffer only, untreated cells (n = 36), light blue line: buffer only, UV-treated cells (n = 60), black line: H1, untreated cells, green line: H1, UV-treated cells. The graph shows that upon UV irradiation, chromatin escape occurs significantly faster in H1-exposed cells than in untreated control cells, with a 50% loss after 2.5 min. The finding supports the importance of caspase-3 activity in H1-mediated NEBD.
(TIF)

Movie S1 **Microinjection of dextran 40 (yellow) and antibodies (violet) in U2OS cells (negative control, bleach corrected).**
(AVI)

Movie S2 **Microinjection of dextran 40 (yellow), antibodies (violet) and ∼100 H1 in U2OS cells (bleach corrected).**
(AVI)

Movie S3 **Microinjection of dextran 40 (yellow), antibodies (violet) and Ca^++^ into U2OS cells.** The final intracellular Ca^++^ concentration was 7 mM corresponding to the concentration found in the ER (bleach corrected).
(MOV)
