# Supplementary figures and images for: Parvoviruses Cause Nuclear Envelope Breakdown by Activating Key Enzymes of Mitosis
Source: PLoS Pathog. 2013 Oct 31;9(10):e1003671. doi: 10.1371/journal.ppat.1003671 (PMC3814971; doi:10.1371/journal.ppat.1003671)

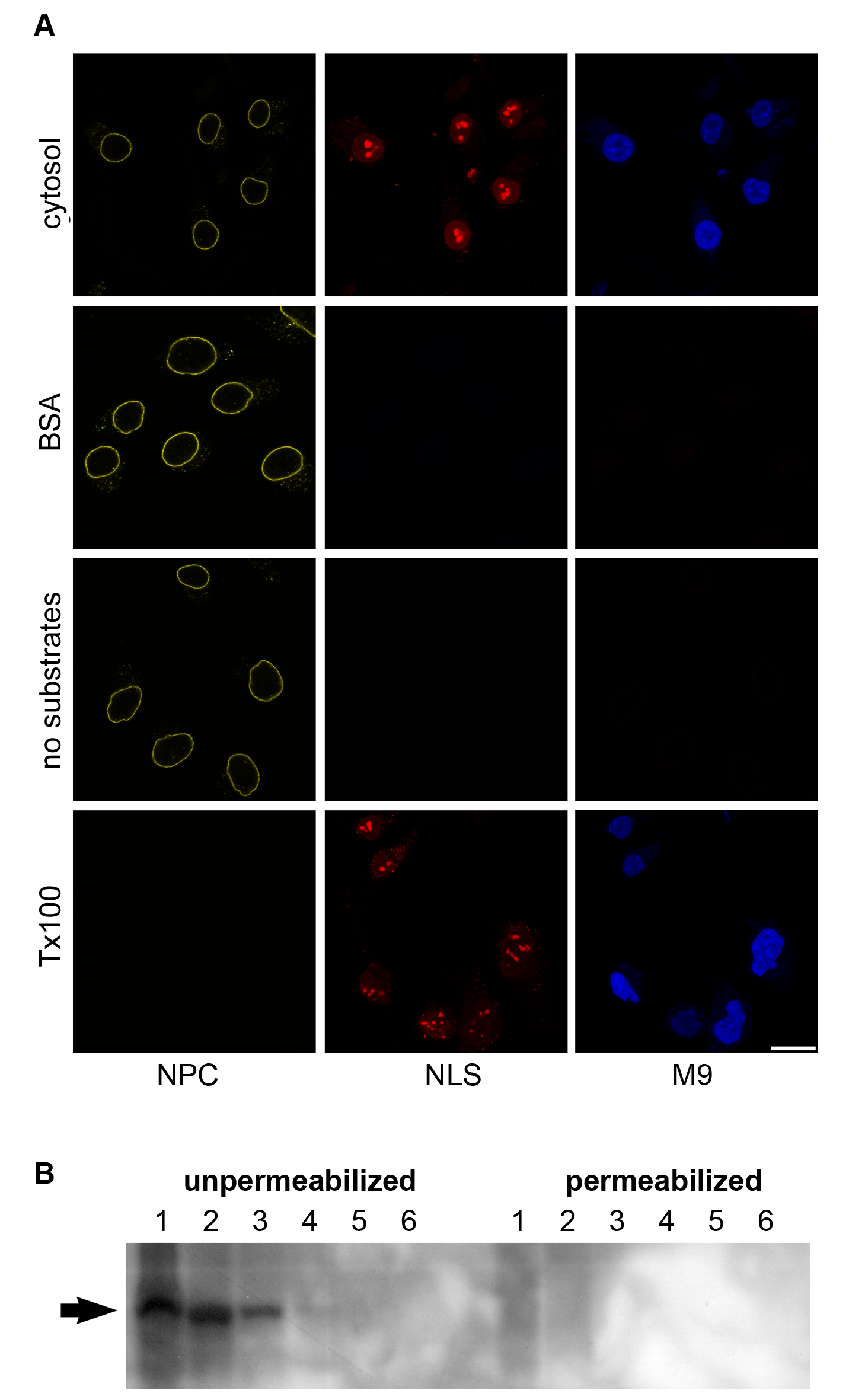

Supplement: Figure S1 — Permeabilized HeLa cells do not contain significant amounts of soluble cytosolic proteins. A. Nuclear import assay of karyophilic substrates, visualized by LSM. NPC: indirect fluorescence of the NE using mAb414, which binds to different proteins of the nuclear pore; M9: direct fluorescence of nuclear M9-Alexa647-BSA, NLS: direct fluorescence of nuclear NLS-Alexa594-BSA conjugate. First row: permeabilized cells+both karyophilic cargos (M9-Alexa647-BSA and NLS-Alexa594-BSA)+exogenous cytosolic extract. Second row: as before but in the absence of cytosol. Third row: permeabilized cells+exogenous cytosolic extract without cargos. Fourth row: import as in the first row, followed by treatment with Tx-100 to remove the membrane. The figure shows that no visible active nuclear import occurs in permeabilized HeLa cells after permeabilization and washing. Replacement of the cytosolic factors by cytosol reconstitutes the nuclear import capacity of the nuclei. Lower row: control reaction to test whether permeabilization of the nuclear membrane alone cause escape of M9-Alexa647-BSA from the nucleus. The nuclei of the digitonin-permeabilized HeLa cells were loaded by the karyophilic cargos in the presence of cytosolic extract as in the upper two rows, then washed and incubated with 0.5% Tx-100 at RT until the nuclei lost contact from the cover slip. The nuclei were sedimented onto collagenized cover slips, fixed and the NPC was stained by indirect immune fluorescence. The loss of NPC stain indicates that the Tx-100 treatment removed the NE including the integrated NPCs. Bar = 25 µm. B. Immune detection of α tubulin after SDS PAGE in unpermeabilized and Digitonin-permeabilized cells. The arrow indicates the migration of α tubulin (66 kDa). The treatment of the cells is indicated on top of the figure 1. Lysate of 2.5×10^6^ HeLa cells, 2. 1∶4, 3. 1∶16, 4. 1∶64, 5. 1∶ 256, 6. 1∶1024 dilutions. The blot shows that upon permeabilization and washing on ice – which depolymerizes microtubul [file ppat.1003671.s001.tif]

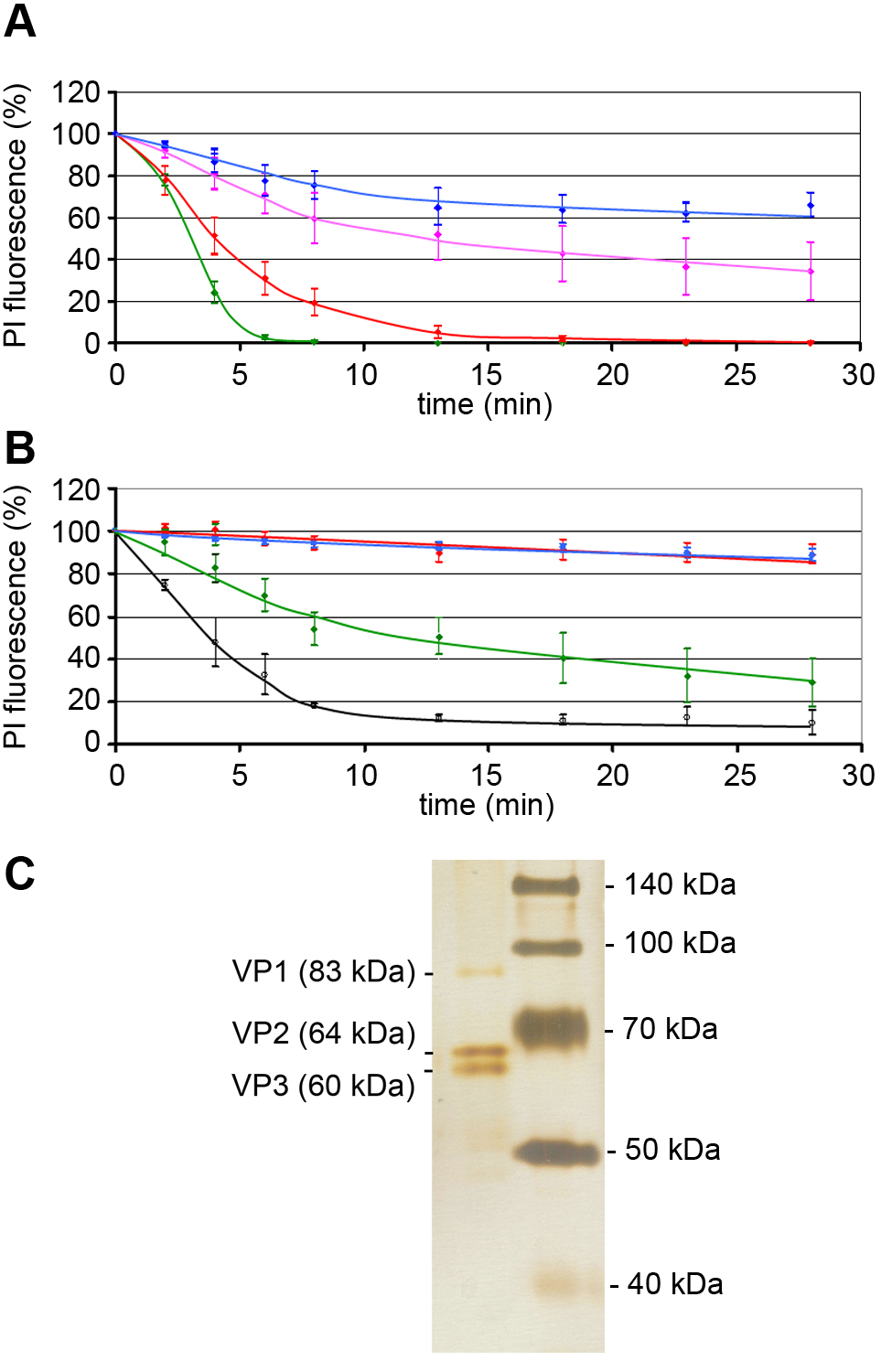

Supplement: Figure S2 — H1-mediated NEBD is dose-dependent but independent upon contaminating factors. Quantifications of the PI fluorescences in permeabilized HeLa cell nuclei with the mean values and the 95% confidence intervals (bars). X-axes: time in min; y axes: relative PI fluorescence. A. Dose-response curve of chromatin escape. Blue: 37.5 genome-containing H1 particles permeabilized cell (n = 12), pink: 150 H1 particles permeabilized cell (n = 9), red: 300 H1 particles permeabilized cell (n = 10), green: 600 H1 particles permeabilized cell (n = 6). B. Loss of chromatin is independent upon H1 preparation method. Red line: buffer only (n = 5), black: 300 iodixanol-purified H1 permeabilized HeLa cell (n = 8), green: 300 CsCl gradient-purified H1 permeabilized cell (n = 5), cyan: iodixanol MOCK-purification, in which uninfected cells were subjected to the purification protocol used for H1 (n = 7). Although NEBD was visible for both H1 preparations but not for the MOCK control the CsCl gradient preparation showed a slower kinetic. This is in agreement with a lower infectivity of PV upon this purification protocol (not shown). C. Silver stain of iodixanol gradient purified H1 after SDS PAGE. The MW of the marker proteins are shown on the right, the H1 proteins are indicated on the left of the gel. The silver stain exhibits the three structural proteins of H1, VP1, VP2 and VP3 with their characteristic MW. Three faint additional bands are visible with a MW of approximately 50 kDa. The Western blot confirms that these bands are reactive for the anti H1 antibody (not shown). The purity of the CsCl gradient purified capsids is shown elsewhere [1] showing exclusively VP1, VP2 and VP3. In summary the data show that PV-mediated NEBD is not caused by contaminating factors of the H1 preparation. (TIF) [file ppat.1003671.s002.tif]

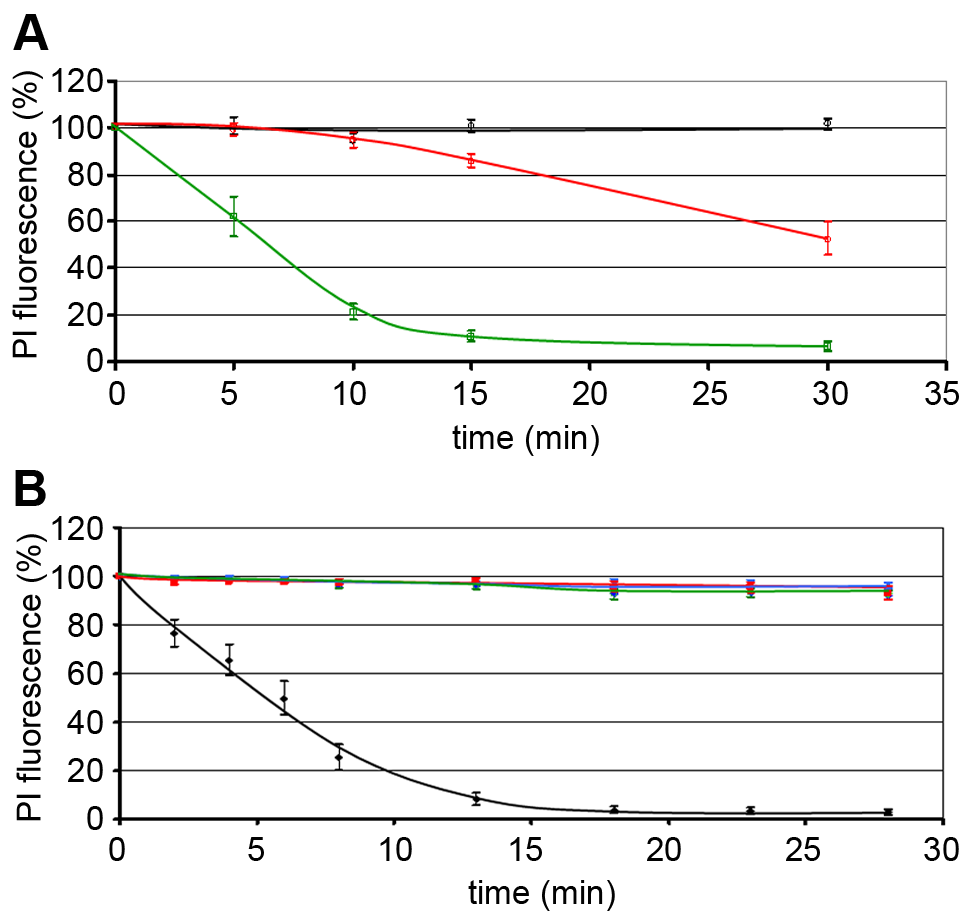

Supplement: Figure S3 — H1 mediated NEBD is temperature and energy dependent. A. Temperature-dependence of chromatin escape using 300 H1 per permeabilized HeLa cell. The graph depicts the mean values and the 95% confidence intervals (bars) as in Fig. S2. Black line: buffer only at 37°C (n = 7), red line: H1 at RT (n = 9), green line H1 at 37°C (n = 8). The graph shows that H1 at RT causes a 50% loss of PI fluorescence occurred after 30 min, while a 50% reduction at 37°C occurred after 5 min. B. Energy-dependence of H1-mediated chromatin escape. Red line: buffer only (n = 34), green line: buffer with hexokinase/glucose, which depletes ATP and GTP from the permeabilized cells (n = 32), blue line: hexokinase/glucose with 300 H1 per permeabilized cell (n = 27), black line: 300 H1 per permeabilized cell (n = 23). The two graphs show that H1-mediated NEBD is an energy- and temperature-dependent process, indicating the need of enzymatic activities. (TIF) [file ppat.1003671.s003.tif]

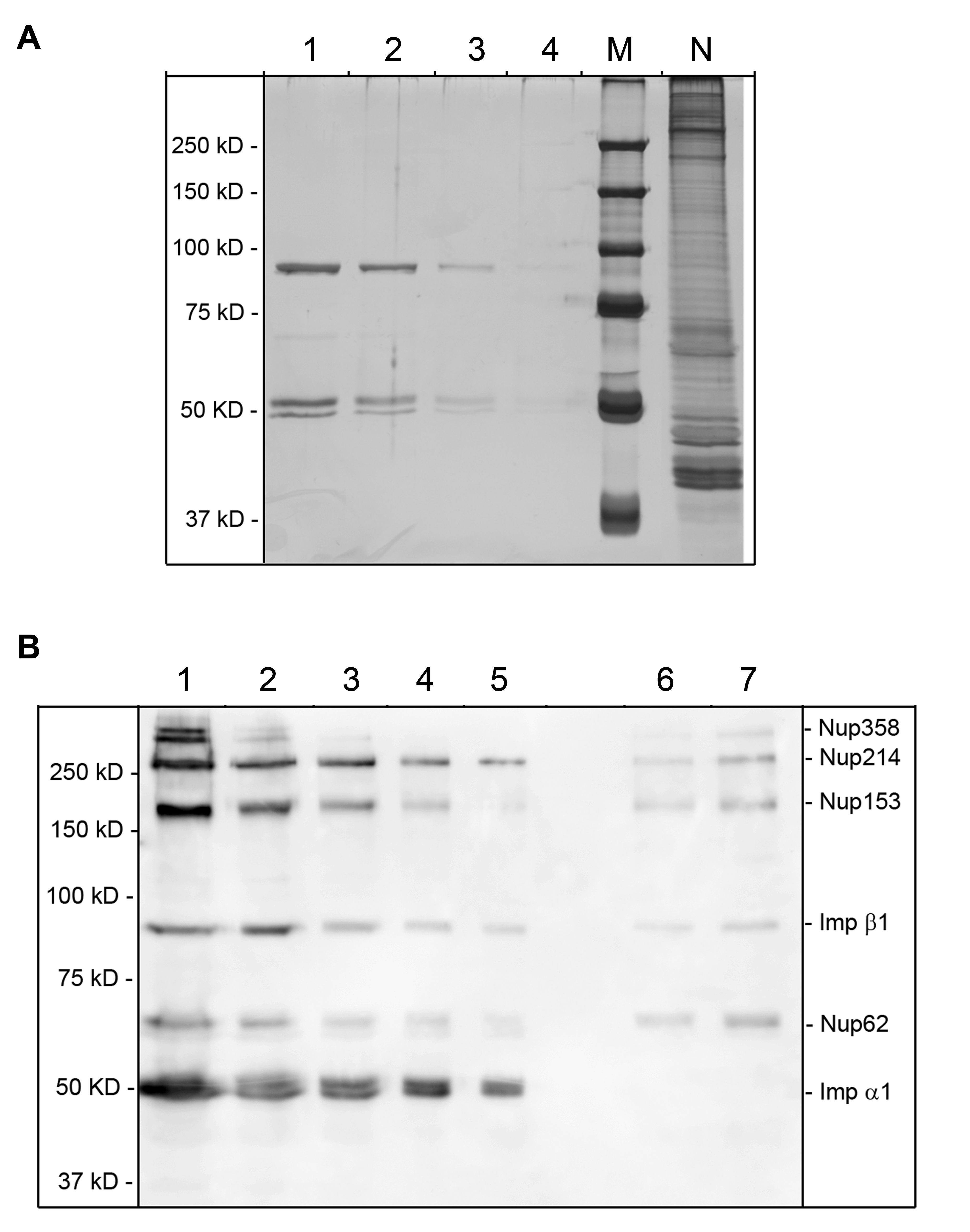

Supplement: Figure S4 — Analysis of the Nup preparation. A. Silver stain after SDS PAGE. The MW of the marker is given on the left. 1: 250 ng, 2: 125 ng, 3: 62.5, 4: 31.3 ng of importin α and importin β each. M: MW marker, N: nine µg (total protein) of the Nup preparation. B. Western blot of the lysates from intact HeLa cells and of the Nup preparation after SDS PAGE using mAbs against importin α, importin β and an antibody against Nups 358, 214, 153 and 62 (mAb414). The MW of the marker is given on the left, The migration of the different proteins is indicated on the right. 1: 5×10^6^ cells, 2: 1×10^6^ cells, 3: 2×10^5^ cells, 4: 4×10^4^ cells, 5: 8×10^3^ cells. 6: 18 µg (total protein) of the Nup preparation, 7: 36 µg. Based on Nup62, which is an internal protein of the NPC, it can be concluded that 36 µg of the Nup preparation contain the Nups of 2×10^6^ cells. The importin α signal is however much weaker than in 8×10^3^ cells indicating a reduction of more than 500fold. Importin β was reduced at least 50fold. The relative strength of the Nup bands changed between intact cells and the Nup preparation indicating that the NPC were separated into isolated Nups. (TIF) [file ppat.1003671.s004.tif]

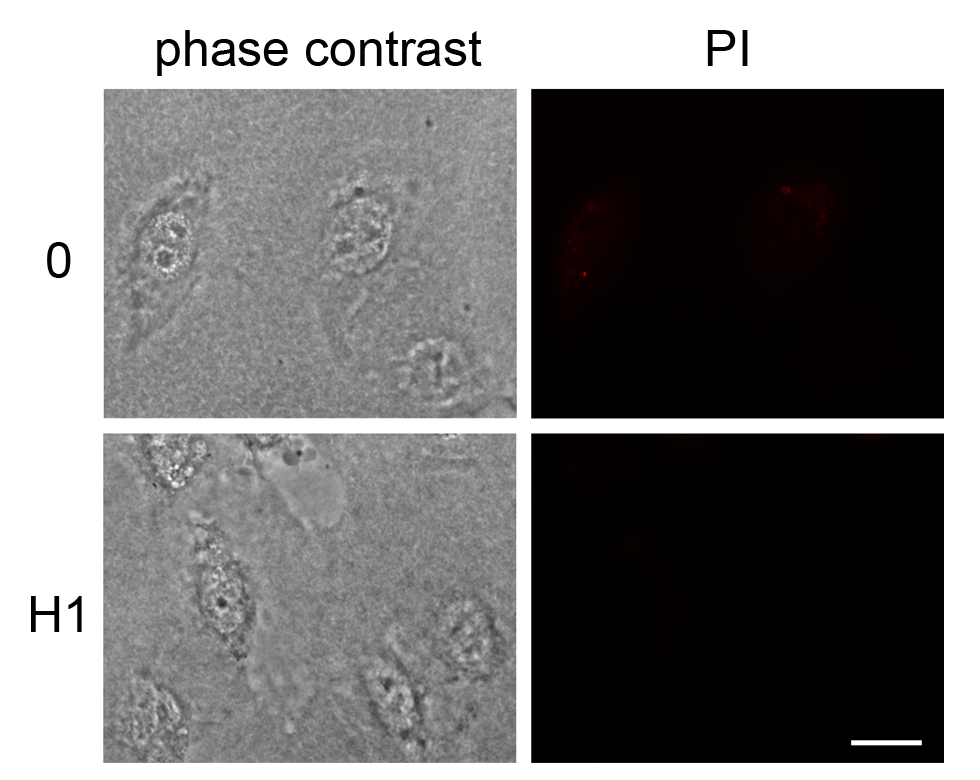

Supplement: Figure S5 — H1 does not degrade the plasma membrane of HeLa cells. Wide field microscopy of HeLa cells. Left: phase contrast, right: PI fluorescence. H1: Three hundred H1, in a concentration corresponding to the conditions used in cells with permeabilized cells were added for 15 min at 37°C in medium mixed with PI. . ∅: control with PI/medium. As PI is cell impermeable the absence of significant stain indicates that the cells stayed intact and that H1 do not degrade membranes unspecifically. Bar = 10 µm. (TIF) [file ppat.1003671.s005.tif]

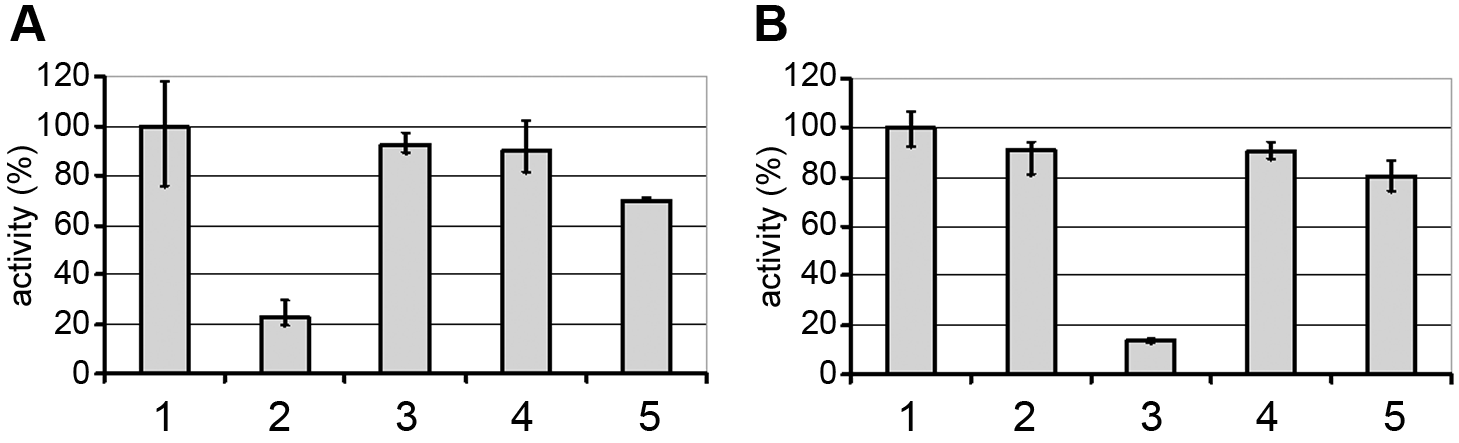

Supplement: Figure S6 — Specificity of inhibitors against PKC, cdk-2 and caspase-3. A. Mixture of PKC isoforms (α,β,γ,δ,ε,μ,θ,ζ): 1. no inhibitor, 2. 10 µM H89, 3. 50 µM roscovitine, 4. 50 µM zVAD-fmk. Y-axis: relative activity in % of the non-inhibited control. The bars indicate the range of 3 independent assays B. Cdk-2. Columns as in A. The panels show that PKC and cdk-2 were inhibited to ∼20% by the corresponding inhibitor but not affected by the inhibitors of the other protein kinase. Thapsigargin showed a slight inhibition of 20%. Thapsigargin was removed prior to permeabilization and PV addition, excluding significant cross inhibition. In summary the figures show that there was no significant unspecific inhibition of the inhibitors regarding to the tested enzymes. (TIF) [file ppat.1003671.s006.tif]

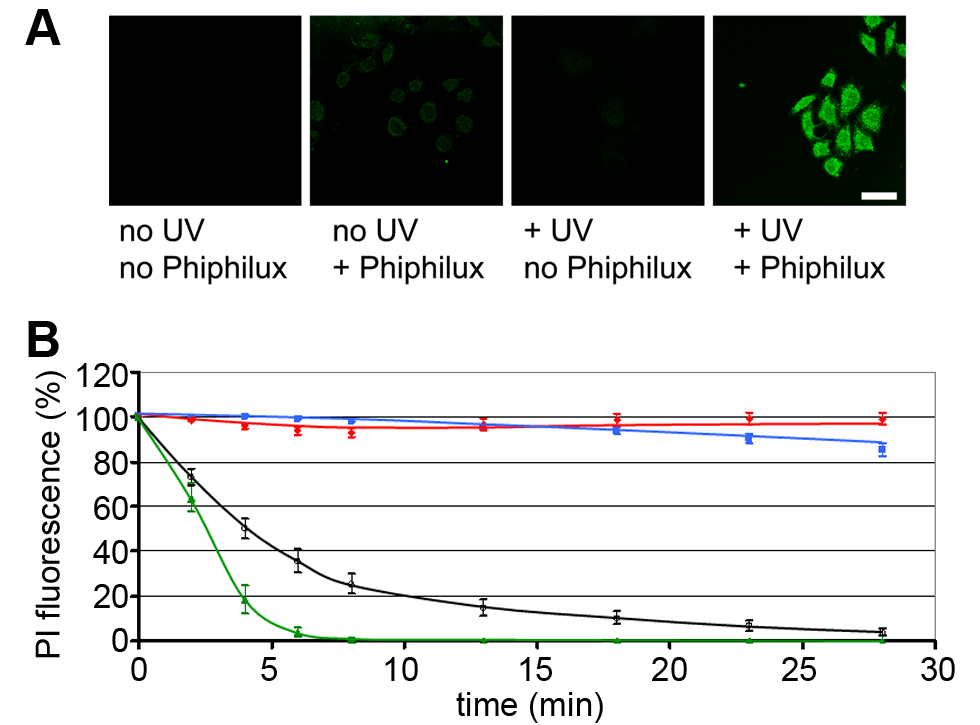

Supplement: Figure S7 — Caspase-3 activation increases the speed of H1-mediated chromatin escape. A. Phiphilux fluorescence of HeLa cells 1 h after irradiation with 9,000 µW/cm2 UV light (λ = 312 nm). Bar = 20 µm. The figure shows that UV light strongly induced caspase-3 activity mainly in the cytoplasm but also intranuclear. B. H1-mediated chromatin escape of permeabilized UV-irradiated cells by 300 H1 permeabilized cell. The graphs show quantifications of the PI fluorescences with the mean values and the 95% confidence intervals (bars). Red line: buffer only, untreated cells (n = 36), light blue line: buffer only, UV-treated cells (n = 60), black line: H1, untreated cells, green line: H1, UV-treated cells. The graph shows that upon UV irradiation, chromatin escape occurs significantly faster in H1-exposed cells than in untreated control cells, with a 50% loss after 2.5 min. The finding supports the importance of caspase-3 activity in H1-mediated NEBD. (TIF) [file ppat.1003671.s007.tif]
